# Pareto optimization in algebraic dynamic programming

**DOI:** 10.1186/s13015-015-0051-7

**Published:** 2015-07-07

**Authors:** Cédric Saule, Robert Giegerich

**Affiliations:** Faculty of Technology and the Center for Biotechnology, Bielefeld University, Bielefeld, Germany

**Keywords:** Pareto optimization, Dynamic programming, Algebraic dynamic programming, RNA structure, Sankoff algorithm

## Abstract

Pareto optimization combines independent objectives by computing the Pareto front of its search space, defined as the set of all solutions for which no other candidate solution scores better under *all* objectives. This gives, in a precise sense, better information than an artificial amalgamation of different scores into a single objective, but is more costly to compute. Pareto optimization naturally occurs with genetic algorithms, albeit in a heuristic fashion. Non-heuristic Pareto optimization so far has been used only with a few applications in bioinformatics. We study exact Pareto optimization for two objectives in a dynamic programming framework. We define a binary Pareto product operator ${*}_{\text {Par}}$ on arbitrary scoring schemes. Independent of a particular algorithm, we prove that for two scoring schemes *A* and *B* used in dynamic programming, the scoring scheme $A {*}_{\text {Par}}B$ correctly performs Pareto optimization over the same search space. We study different implementations of the Pareto operator with respect to their asymptotic and empirical efficiency. Without artificial amalgamation of objectives, and with no heuristics involved, Pareto optimization is faster than computing the same number of answers separately for each objective. For RNA structure prediction under the minimum free energy versus the maximum expected accuracy model, we show that the empirical size of the Pareto front remains within reasonable bounds. Pareto optimization lends itself to the comparative investigation of the behavior of two alternative scoring schemes for the same purpose. For the above scoring schemes, we observe that the Pareto front can be seen as a composition of a few macrostates, each consisting of several microstates that differ in the same limited way. We also study the relationship between abstract shape analysis and the Pareto front, and find that they extract information of a different nature from the folding space and can be meaningfully combined.

**Electronic supplementary material:**

The online version of this article (doi:10.1186/s13015-015-0051-7) contains supplementary material, which is available to authorized users.

## Background

In combinatorial optimization, we evaluate a search space *X* of solution candidates by means of an objective function $\psi$. Generated from some input data of size *n*, the search space *X* is typically discrete and has size $O(\alpha ^n)$ for some $\alpha$. Conceptually, as well as in practice, it is convenient to formulate the objective function as the composition of a *choice function*$\varphi$ and a *scoring function*$\sigma$, $\psi = \varphi \circ \sigma$, computing their composition as $(\varphi \circ \sigma )(X) = \varphi (\{\sigma (x)|x \in X\})$ for the overall solution. The most common form of the objective function $\psi$ is that $\sigma$ evaluates each candidate to a score (or cost) value, and $\varphi$ chooses the candidate which maximizes (or minimizes) this value. One or all optimal solutions can be returned, and with little difficulty, we can also define $\varphi$ to compute all candidates within a threshold of optimality. This scenario is the prototypical case we will base our discussion on. However, it should not go unmentioned that there are other, useful types of “choice” functions besides maximization or minimization, such as computing score sums, full enumeration of the search space, or stochastic sampling from it.

Multi-objective optimization arises when we have several criteria to evaluate our search space. Scanning the pizza space of our home town, we may be looking for the largest pizza, the cheapest, or the vegetarian pizza with the richest set of toppings. When we use these criteria in combination, the question arises exactly how we combine them.

Let us consider two objective functions $\psi _1 = \varphi _{1}\circ \sigma _1$ and $\psi _2 = \varphi _{2} \circ \sigma _2$ on the search space *X* and let us define different variants of an operator $*$ to designate particular techniques of combining the two objective functions.

- *Additive combination*$(\psi _1\,\, {*}_{\text {+}}\,\,\psi _2)$ optimizes over the sum of the candidates scores computed by $\varphi _1$ and $\varphi _2$. This is a natural thing to do when the two scores are of the same type, and optimization goes in the same direction, i.e. $\varphi _1 = \varphi _2 =: \varphi$; we define
  1 $$\begin{aligned} \psi _1\,\, {*}_{\text {+}}\,\,\psi _2 = \varphi \circ (\sigma _1 + \sigma _2). \end{aligned}$$
  In fact, this recasts the problem in the form of a single objective problem with a combined scoring function. This applies, e.g. for real costs (money), which sum up in the end no matter where they come from. Gotoh’s algorithm for sequence alignment under an affine gap model can be seen as an instance of this combination [1]. It minimizes the score sum of character matches, gap openings and gap extensions. Jumping alignments are another example [2]. They align a sequence to a multiple sequence alignment. The alignment always chooses the alignment row that fits best, but charges a cost for jumping to another row. Jump cost and regular alignment scores are balanced based on test data. However, often it is not clear how scores should be combined, and researchers resort to more general combinations.
- *Parametrized additive combination*$(\psi _1\,\, {*}_{\text {+}\lambda }\,\,\psi _2)$ is defined as
  2 $$\begin{aligned} \psi _1\,\, {*}_{\text {+}\lambda }\,\,\psi _2 = \varphi \circ (\lambda \sigma _1 + (1-\lambda )\sigma _2), 0 \le \lambda \le 1. \end{aligned}$$
  Here the extra parameter signals that there is something artificial in the additive combination of scores, and the $\lambda$ is to be trained from data in different application scenarios, or left as a choice to the user of the approach. Such functions are often used in bioinformatics  [3–7]. For example, the Sankoff algorithm scores joint RNA sequence alignment and folding by a combination of base pairing ($\psi _1$) and sequence alignment ($\psi _2$) score [3]. RNAalifold scores consensus structures by a combination of free energy ($\sigma _1$) and covariance ($\sigma _2$) scores [8]. Covariance scores are converted into “pseudo-energies”, and the parameter $\lambda$ controls the relative influence of the two score components. This combination often works well in practice, but a pragmatic smell remains. Returning to our earlier pizza space example: It does not really make sense to add the number of toppings to the size of the pizza, or subtract it from the price, no matter how we choose $\lambda$. In a way, the parameter $\lambda$ manifests our discomfort with this situation.
- *Lexicographic combination*$(\psi _1\,\, {*}_{\text {lex}}\,\,\psi _2)$ performs optimization on pairs of scores of potentially different type, not to be combined into a single score.
  3 $$\begin{aligned} (\psi _1\,\, {*}_{\text {lex}}\,\,\psi _2)(X) = (\varphi _1,\varphi _2)(\{(\sigma _1(x),\sigma _2(x)) | x \in X\}), \end{aligned}$$
  where $(\varphi _1,\varphi _2)$ optimizes lexicographically on the score pairs $(\sigma _1(x),\sigma _2(x))$. With the lexicographic combination, we define a primary and a secondary objective, seeking either the largest among the cheapest pizzas, or the cheapest among the largest—certainly with different outcomes. This is very useful, for example, when $\varphi _1$ produces a large number of co-optimal solutions. Having a secondary criterion choose from the co-optimals is preferable to returning an arbitrary optimal solution under the first objective, maybe even unaware that there were alternatives.
  Lexicographic and parameterized additive combination are incomparable with respect to their scope. ${*}_{\text {lex}}$ can combine scoring schemes of different types, but cannot optimize a sum of two scores even when they have the same type. ${*}_{\text {+}\lambda }$ does exactly this. Only when both scores have the same type, there may be a choice of $\lambda$ big enough such that ${*}_{\text {+}\lambda }$ emulates ${*}_{\text {lex}}$.
- *Pareto combination*$(\psi _1\,\, {*}_{\text {Par}}\,\,\psi _2)$ must be used in the case when there is no meaningful way to combine or prioritize the two objectives. It may also be useful and more informative in the previous scenarios, producing a set of “optima” and letting the user decide the balance between the two objectives *a posteriori*.
  Pareto optimality is defined via (non-)domination. An element $(a,b) \in (A \times B)$*dominates* another one, if it is strictly better in one dimension, and not worse in the other. The solution set one computes is the *Pareto front* of $\{(\sigma _1(x),\sigma _2(x))|x\in X\}$. Taking $\varphi _1$ and $\varphi _2$ as maximization, the Pareto front operator ${\mathbf{pf}}$ is defined on subsets *S* of $A\times B$, ordered by $>_A$ and $>_B$, respectively, as follows:
  4 $$\begin{aligned} {\mathbf{pf}} (S)&= \{(a,b) \in S ~|~ \not \exists (a',b') \in {{S}{\setminus} \{(a,b)\}} \nonumber \\&\quad {\text { such that }} (a',b') {\text { dominates }} (a,b) \}. \end{aligned}$$
  A set without dominated elements is called a Pareto set. Naturally, every subset of a Pareto set is a Pareto set, too. We define the Pareto combination as
  5 $$\begin{aligned} (\psi _1 \,\,{*}_{\text {Par}}\,\,\psi _2)(X) = {\mathbf{pf}} \{(\sigma _1(x),\sigma _2(x)) | x \in X\}. \end{aligned}$$
  The Pareto combination ${*}_{\text {Par}}$is more general than both ${*}_{\text {+}\lambda }$ and ${*}_{\text {lex}}$. This is obvious for ${*}_{\text {lex}}$, and is also obvious for ${*}_{\text {+}\lambda }$ when the two scores are of different type. It even holds for ${*}_{\text {+}\lambda }$ when they have the same type, but becomes more subtle. It can be shown that ${*}_{\text {Par}}$ produces all optima that can be produced by ${*}_{\text {+}\lambda }$ for some $\lambda$, but there can also be others. See Theorem 3.1, and we also refer the reader to the careful treatment of this intriguing issue in Schnattinger’s thesis [9].

The combinations ${*}_{\text {+}}$ and ${*}_{\text {+}\lambda }$ are common practice and merit no theoretical investigation, as they reduce the problem to the single objective case. The lexicographic combination ${*}_{\text {lex}}$ has been studied in detail in [10]. Aside from its obvious use with primary and secondary objectives, ${*}_{\text {lex}}$ has an amazing variety of applications when of one the two objectives does not perform optimization, but enumeration or summation (cf. above). Furthermore, when $\sigma _1$ computes a classification attribute from the candidates and $\varphi _1$ is the identity, it gives rise to the method of *classified* dynamic programming used, e.g. in probabilistic RNA shape analysis [11].

Here, we will take a deeper look into Pareto optimization, which has been used in bioinformatics mainly in a heuristic fashion. It naturally arises with the use of genetic algorithms. They traverse the search space improving candidates in a certain dimension, returning a solution when (say) $\sigma _1(x)$ can no longer be increased without decreasing $\sigma _2(x)$. A genetic algorithm uses different starting points and produces a heuristic subset of the Pareto front of the search space. This approach was used by Zhang et al. [12], who compute the Pareto front to solve a multiple sequence alignment problem. *Cofolga2mo* [13] is a structural RNA sequence aligner based on a multi-objective genetic algorithm. *Cofolga2mo* does not look strictly at the Pareto optimal solutions, but produces a heuristic subset of the (larger) set of “weak” Pareto optimal solutions. The objective function is computed on similarity sequence score and consensus structure score (under the base-pairing probabilities model). Only the score of the consensus structure is given, the structure itself is not shown.

A similar approach was used by Taneda [14] to solve the inverse RNA folding problem by computing the Pareto front between the folding energy in the first dimension and the similarity score to the target in the second dimension. In [15], the authors compute the Pareto front for multi-class gene selection. They use a genetic algorithm to avoid the statistics aggregation in gene expression, which could lead to the “siren pitfall” issue [16] when using ${*}_{\text {+}\lambda }$.

Let us now turn to non-heuristic cases of Pareto optimization. A family of monotonous operators in preordered and partially ordered sets in dynamic programming were defined in [17]. The author showed the principle of optimality applies to the maximal return in the case of Markovian processes. Such processes evolve stochastically over time and do not consume any input. We are aware of only a few cases where Pareto optimization has been advocated within a dynamic programming approach. It was used by Getachew et al. [18] to find the shortest path in a network given different time cost/functions, computing the Pareto front. The Pareto-Optimal Allocation problem was solved with dynamic programming by Sitarz [19]. In the field of bioinformatics, Schnattinger et al. [20, 21] advocated Pareto optimization for the Sankoff problem. Their algorithm computes $(\psi _1\,\, {*}_{\text {Par}}\,\,\psi _2)$, where $\psi _1$ optimizes a sequence similarity score and $\psi _2$ optimizes base pair probabilities in the joint folding of two RNA sequences.

Libeskind-Hadas et al. [22] used an exact Pareto optimisation by using dynamic programming to compute reconciliation trees in phylogeny. They introduce two binary operators $\oplus$ and $\otimes$ which stand respectively for *set union* and *set cartesian product*, both followed by a Pareto filtration for their specific problem. They optimized *A*$\otimes$*B*, where *A* and *B* are sets, by sorting *A* and *B* in lexicographical order and keeping the resulting Pareto list sorted.

Pareto optimization in a dynamic programming approach raises four specific questions, which we will address in the body of this article.

(i) Does the Pareto combination of two objectives satisfy Bellman’s principle of optimality, the prerequisite for all dynamic programming ("Pareto optimization in ADP")?
(ii) How to compute Pareto fronts both efficiently and incrementally, when proceeding from smaller to larger sub-problems ("Implementation)?
(iii) What is the empirical size of the Pareto front, compared to its expected size ("Applications")?
(iv) What observations can de drawn from a computed Pareto front in a concrete application ("Applications")?

Heretofore, the issues (i) and (ii) had to be solved ad-hoc with every approach employing Pareto optimization. Motivated by and generalizing on the work by Schnattinger et al., we strive here for general insight in the use of Pareto optimization within dynamic programming algorithms. To maintain a well-defined class of dynamic programming algorithms to which our findings apply, we resort to the framework of* algebraic* dynamic programming (ADP) [23].

Here is a short preview of our findings: we can prove, in a well circumscribed formal setting, that the Pareto combination preserves Bellman’s principle of optimality (Theorem 3.3). Thus, it is amenable to implementation in a dynamic programming framework such as ADP as a “single keystroke” operation. We show that while the search space size is typically exponential, we can expect Pareto fronts of linear size. This is confirmed empirically by our implementations of ${*}_{\text {Par}}$ (covering several algorithmic variants), and we observe that this is actually more efficient that producing a similar number of (near-optimal) solutions with other means. Finally, using our implementations in the field of RNA structure prediction in some (albeit preliminary) experiments, we find that a small Pareto front in joint alignment and folding may be indicative of a homology relationship, and elucidate differences in the MFE and MEA scoring schemes for RNA folding that could not be observed before.

## Pareto sets: properties and algorithms

We introduce Pareto sets together with some basic mathematical properties and algorithms ("Pareto sets and the Pareto front operator", "Worst case and expected size of Pareto fronts"). We restrict our discussion to Pareto optimization over value pairs, rather than vectors of arbitrary dimension. Pareto optimization in arbitrary dimension is shortly touched upon in our concluding section.

The operations on Pareto sets that arise in a dynamic programming framework are threefold: taking the Pareto front of a set of sub-solutions ("Computing the Pareto front", "Pareto operator complexity, revisited"), joining alternative solution sets ("Pareto merge in linear time"), and computing new solutions from smaller subproblems by the application of scoring functions ( "Pareto set extension").

### Pareto sets and the Pareto front operator

We start from two sets *A* and *B* and their Cartesian product $C = A \times B$. The sets *A* and *B* are totally ordered by relations $>_\text {A}$ and $>_\text {B}$, respectively. This induces a partial *domination* relation $\succ$ on *C* as follows. We have $(a,b) \succ (a',b')$ if $a >_\text {A} a'$ and $b \ge _\text {B} b'$, or $a \ge _\text {A} a'$ and $b >_\text {B} b'$. In words, the dominating element must be larger in one dimension, and not smaller in the other. In $X \subseteq C$, an element is *dominant* iff there is no other element in *X* that dominates it. A set without dominated elements is a *Pareto set*. We can restate Eq. (4) in words as: The *Pareto front* of *X*, denoted ${\mathbf{pf}} (X)$, is the set of all dominant elements in *X*. The definition of ${\mathbf{pf}}$ actually depends on the underlying total orders, and we should write more precisely ${\mathbf{pf}} _{>_\text {A},>_\text {B}}$, but for simplicity, we will suppress this detail until it becomes relevant.

The following properties hold by definition and are easy to verify:

6 $$\begin{aligned} {\mathbf{pf}} (X)\subseteq & {} X \end{aligned}$$

7 $$\begin{aligned} {\mathbf{pf}} (X) = \emptyset\iff & {} X = \emptyset \end{aligned}$$

8 $$\begin{aligned} {\mathbf{pf}} (\mathbf pf (X))= & {} {\mathbf{pf}} (X) \end{aligned}$$

9 $$\begin{aligned} {\mathbf{pf}} (X \cap Y) \, {\supseteq } \, {\mathbf{pf}} (X) \cap {\mathbf{pf}} (Y) \end{aligned}$$

Note that ${\mathbf{pf}}$ is not monotone with respect to $\subseteq$. Idempotency of ${\mathbf{pf}}$ (Eq. 8) justifies the alternative definition: A set $X \subseteq C$ is a *Pareto set* if ${\mathbf{pf}} (X) = X$.

Algorithmically, we represent sets as lists, without duplicate elements. If a list represents a Pareto set, we call it a *Pareto list*.

A *sorted Pareto list*, by definition, is sorted lexicographically under $(>_\text {A},>_\text {B})$ in decreasing order. Naturally, on sorted lists, we can perform certain operations more efficiently, which must be balanced against the effort of keeping lists sorted.

The intersection of two Pareto sets is a Pareto set because it is a subset of a Pareto set by (9). This does not apply for Pareto set union, as elements in one Pareto set may be dominated by elements from the other. Therefore, we define the *Pareto merge* operation

10 $$\begin{aligned} A \mathop {\vee }\limits ^{p}B := {\mathbf{pf}} (A \cup B) \end{aligned}$$

Clearly, $\mathop {\vee }\limits ^{p}$ inherits commutativity from $\cup$.

#### **Observation 1**

(Pareto merge associativity)

11 $$\begin{aligned} (A \mathop {\vee }\limits ^{p}B) \mathop {\vee }\limits ^{p}C = A \mathop {\vee }\limits ^{p}(B \mathop {\vee }\limits ^{p}C) \end{aligned}$$

We show that both sides are equal to $U:={\mathbf{pf}} (A \cup B \cup C)$.

Let $x \in U$. Clearly, $x \in A \cup B\cup C$, and $\not \exists x' \in A \cup B \cup C$ such that $x' \succ x$. This holds if and only if there is no such $x'$ in $A\mathop {\vee }\limits ^{p}B$, nor in *C*, which is equivalent to $x \in (A \mathop {\vee }\limits ^{p}B) \mathop {\vee }\limits ^{p}C$. $A \mathop {\vee }\limits ^{p}(B \mathop {\vee }\limits ^{p}C) = U$ follows by a symmetric argument. $\Box$

As a consequence, we can simply write $A \mathop {\vee }\limits ^{p}B \mathop {\vee }\limits ^{p}C$. Note that in practice, it may well make a difference in terms of efficiency whether we compute a three-way Pareto merge as $(A \mathop {\vee }\limits ^{p}B) \mathop {\vee }\limits ^{p}C$ or as ${\mathbf{pf}} (A \cup B \cup C)$.

### Worst case and expected size of Pareto fronts

In combinatorial optimization, the search space is typically large, but finite. This allows for some statements about the maximal and the expected size of a Pareto front.

#### **Observation 2**

 (Sorted Pareto lists) A Pareto list sorted on the first dimension based on $>_A$ (i) is also sorted lexicographically by $(>_A,>_B)$ in decreasing order, and at the same time (ii) is sorted lexicographically in *increasing* order based on $(>_B,>_A)$.

This is true because when the list *l* is a Pareto list and $(a,b) \in l$, there can be no other element $(a,b')$ with $b \ne b'$. Because $>_\text {B}$ is a total order, one of the two would dominate the other. Therefore, (i) the overall lexicographic order is determined solely by $>_\text {A}$, and (ii) looking at the values in the second dimension alone, we find them in increasing order of $>_\text {B}$. $\Box$

This implies a worst-case observation on the size of Pareto fronts over discrete intervals:

#### **Observation 3**

 (Worst case size of Pareto set) If *A* and *B* are discrete intervals of size *M*, then any Pareto set over $A \times B$ has $N \le M$ elements.

This is true because by Observation 2, each decrease in the first dimension must come with an increase in the second component. $\Box$

#### **Observation 4**

On random sets, the expected size of the Pareto front of a set of size *N* follows the harmonic law [24, 25],

12 $$\begin{aligned} H(N) = \sum _{i=1}^N(1/i). \end{aligned}$$

$$\Box$$

### Computing the Pareto front

We specify algorithms to compute Pareto fronts from unsorted and sorted lists. In our pseudocode, $\varepsilon$ denotes the empty list, *x* : *l* denotes a list with first element *x* and remainder list *l*, and vice versa for *l* : *x*. The arrow $\rightarrow$ indicates term rewriting or state transition.

#### From an unsorted list

 An obvious possibility is to sort the list by an $O(N \log N)$ sorting algorithm, and then compute the Pareto front by one of the algorithms for sorted lists specified below. We call this implementation of the Pareto front operator ${\mathbf{pf}} _\text {sort}$.

However, it is also interesting to combine the two phases. We present a Pareto-version of insertion sort, asymptotically in $O(N^2)$, but potentially fast in practice, because it effectively decreases *N* already during the sorting phase by eliminating dominated elements.

Pareto front operator ${\mathbf{pf}} _\text {isort}$

$$\begin{aligned} \begin{array}{ll} \mathrm{Input{:}} &{} \mathrm{Unsorted \, list.}\\ \mathrm{Output{:}} &{} \mathrm{Sorted \, Pareto \, list \, (in \, decreasing \, order \, according \, to \, the \, first \, component)}. \end{array} \end{aligned}$$

$$\begin{aligned} \begin{array}{lcl} {\mathbf{{pf}}_\text {isort}(\varepsilon )} &{} {\rightarrow } &{} {\varepsilon }\\ {\mathbf{{pf}} _\text {isort}\mathrm{((a,b){:}l)}} &{} {\rightarrow } &{} {\mathrm{into((a,b)}, {\mathbf{pf}}_\text {isort}(l))}\\ \mathrm{into}(\mathrm{(a,b)}, \varepsilon ) &{} \rightarrow &{} {\mathrm{(a,b)}{:}\varepsilon }\\ \mathrm{into}\mathrm{((a,b), (x,y){:}l)} &{} \mathop {\rightarrow }\limits ^{a > x} &{} \mathrm{(a,b){:}remove(b, (x,y){:}l)}\\ \mathrm{into}\mathrm{((a,b), (x,y){:}l)} &{} \mathop {\rightarrow }\limits ^{a = x, b>y} &{} \mathrm{(a,b){:}remove(b, l)}\\ \mathrm{into}\mathrm{((a,b), (x,y){:}l)} &{} \mathop {\rightarrow }\limits ^{a = x, b \le y} &{} \mathrm{(x,y){:}l}\\ \mathrm{into}\mathrm{((a,b), (x,y){:}l)} &{} \mathop {\rightarrow }\limits ^{a < x, b > y} &{} \mathrm{(x,y){:}into((a,b), l)}\\ \mathrm{into}\mathrm{((a,b), (x,y){:}l)} &{} \mathop {\rightarrow }\limits ^{a < x, b \le y} &{} \mathrm{(x,y){:}l}\\ &{}&{}\\ \mathrm{remove}(\mathrm{b}, \varepsilon ) &{} \rightarrow &{} \varepsilon \\ \mathrm{remove}(\mathrm{b, (x,y){:}l}) &{} \mathop {\rightarrow }\limits ^{b<y} &{} \mathrm{(x,y){:}l}\\ \mathrm{remove}(\mathrm{b, (x,y){:}l}) &{} \mathop {\rightarrow }\limits ^{b \ge y} &{} \mathrm{remove(b, l)} \end{array} \end{aligned}$$

The definition of the *remove* function makes use of the inductive property that the list l is already a (sorted) Pareto list, and by our above observation, it is increasing in the second dimension. Hence, we remove one dominated element in each application of the last rule, and terminate when the second rule is applied. All the steps of *remove* are productive in the sense that they reduce the list length for subsequent calls to *into*.

#### From a lexicographically sorted list

From a sorted list, the Pareto front can be extracted in linear time [26]. We describe such an algorithm by a state transition system, which transforms an input and an (initially empty) output list into empty input and the Pareto front as output and call it ${\mathbf{pf}} _\text {lex}.$

$$\begin{aligned} \begin{array}{ll} \mathrm{Input{:}} &{} \mathrm{Sorted \, list.} \\ \mathrm{Output{:}} &{} \mathrm{Sorted \, Pareto \, list.} \end{array} \end{aligned}$$

$$\begin{aligned} \begin{array}{llcll} {\mathbf{{pf}} _\text {lex}(l)} &{} &{} {\rightarrow } &{} \mathrm{l} &{} {\varepsilon } \\ \mathrm{(a,b){:}in} &{} \varepsilon &{} \rightarrow &{} \mathrm {in} &{} {\mathrm{(a,b)}{:}\varepsilon } \\ \mathrm{(a,b){:}in} &{} \mathrm{out{:}(x,y)} &{} \mathop {\rightarrow }\limits ^{y \ge b} &{} \mathrm{in} &{} \mathrm{out{:}(x,y)}\\ \mathrm{(a,b){:}in} &{} \mathrm{out{:}(x,y)} &{} \mathop {\rightarrow }\limits ^{y < b} &{} \mathrm{in} &{} \mathrm{out{:}(x,y){:}(a,b)}\\ \varepsilon &{} \mathrm{out} &{} \rightarrow &{} \mathrm{STOP}&{} \end{array} \end{aligned}$$

Since the input list is shortened by one element in each step, this algorithm runs in *O*(*N*).

#### A smooth Pareto front algorithm for the general case

We can adapt the algorithm ${\mathbf{pf}} _\text {lex}$ to the general case by adding two clauses for elements that appear out of order{:}

We ${\mathbf{pf}} _\text {lex}$ extended by the following rules to obtain ${\mathbf{pf}}_{smooth}$:

$$\begin{aligned} \begin{array}{ll} \mathrm{Input{:}} &{} \mathrm{Unsorted \, list.} \\ \mathrm{Output{:}} &{} \mathrm{Sorted \, Pareto \, list.} \end{array} \end{aligned}$$

$$\begin{aligned} \begin{array}{llcll} {\mathbf{{pf}} _\text {smooth}(\text{l})} &{} &{} {\rightarrow } &{} \mathrm{l} &{} {\varepsilon } \\ \mathrm{(a,b){:}in} &{} \mathrm{out{:}(x,y)} &{} \mathop {\rightarrow }\limits ^{a>x, b \ge y}&{} \mathrm{(a,b){:}in} &{} \mathrm{out}\\ \mathrm{(a,b){:}in} &{} \mathrm{out{:}(x,y)} &{} \mathop {\rightarrow }\limits ^{a>x, b < y}&{} \mathrm{in} &{} \mathrm{up(out, (a,b)){:}(x,y)} \end{array} \end{aligned}$$

where the function *up*(*x*, (*a*, *b*)) inserts the new pair from the low end into the Pareto list *x*:

$$\begin{aligned} \begin{array}{lcl} \mathrm{up}(\varepsilon , \mathrm{(a,b)}) &{} \rightarrow &{} {\mathrm{(a,b)}{:}\varepsilon }\\ \mathrm{up(z{:}(x,y), (a,b))} &{} \mathop {\rightarrow }\limits ^{\mathrm{(a,b)} \succ \mathrm{(x,y)}} &{} \mathrm{up(z, (a,b))}\\ &{} \mathop {\rightarrow }\limits ^{(x,y) \succeq (a,b)} &{} \mathrm{z{:}(x,y)}\\ &{} \mathop {\rightarrow }\limits ^{a>x} &{} \mathrm{up(z, (a,b)){:}(x,y)}\\ &{} \mathop {\rightarrow }\limits ^{a<x} &{} \mathrm{z{:}(x,y){:}(a,b)} \end{array} \end{aligned}$$

Like our first algorithm ${\mathbf{pf}} _\text {isort}$, ${\mathbf{pf}}_\text {smooth}$ handles the general case in quadratic time, but smoothly adapts to sorted lists, becoming the same as ${\mathbf{pf}}_\text {lex}$ when all elements are in order.

#### Unsorted Pareto front computation

Our previous implementations all compute the Pareto front in the form of a sorted list. However, in dynamic programming, solution sets are created in various ways and arise not necessarily sorted, even when sub-solutions are given on sorted order. Hence, it may be attractive to consider an algorithm that does not bother about sorting at all, consumes and produces unsorted lists. We call this variant ${\mathbf{pf}} _\text {nosort}$.

$$\begin{aligned} \begin{array}{ll} \mathrm{Input{:}} &{} \mathrm{Unsorted \, list.} \\ \mathrm{Output{:}} &{} \mathrm{Unsorted \, Pareto \, list.} \end{array} \end{aligned}$$

$$\begin{aligned} \begin{array}{lcl} {\mathbf{pf}}_\text {nosort}(\varepsilon ) &{} \rightarrow &{} \varepsilon \\ {\mathbf{pf}}_\text {nosort}\mathrm{(x{:}y)} &{} \rightarrow &{} \mathrm{into}(\mathrm{x}, {\mathbf{pf}}_\text {nosort}\mathrm{(y)}) \\ &{}&{}\\ \mathrm{into((a,b)}, \varepsilon ) &{} \rightarrow &{} {\mathrm{(a,b)}{:}\varepsilon } \\ \mathrm{into((a,b), (x,y){:}l)} &{} \mathop {\rightarrow }\limits ^{(a,b)\succ (x,y)} &{} \mathrm{into((a,b), l)} \\ \mathrm{into((a,b), (x,y){:}l)} &{} \mathop {\rightarrow }\limits ^{(x,y)\succ (a,b)} &{} \mathrm{(x,y){:}l} \\ \mathrm{into((a,b), (x,y){:}l)} &{} \mathop {\rightarrow }\limits ^{(x,y)\nprec (a,b),(a,b)\nprec (x,y)} &{} \mathrm{(x,y){:}into((a,b), l)} \end{array} \end{aligned}$$

This resembles ${\mathbf{pf}}_\text {isort}$ without sorting the output, and hence the resulting list must always be traversed completely for each element added. Worst case complexity is $O(N^2)$.

### Pareto operator complexity, revisited

For a more detailed complexity analysis of the above algorithms, we must distinguish the size of input and output. In our dynamic programming applications, we will compute ${\mathbf{pf}}(\{f(x,y) | x \in X, y \in Y\})$, where *f* is some local scoring function. If the Pareto sets *X* and *Y* have size *n*, then $\{f(x,y) | x \in X, y \in Y\}$ is of size $n^2$, while the final result can be expected to be smaller again.

For a list of size *N*, the result of ${\mathbf{pf}}$ has size *N* in the worst case. In the expected case, however, output size is *H*(*N*) (Eq. 12), and because $H(N) \approx \ln N$ [24], we can asymptotically treat it as $O(\log N)$. Our observations are summarized in Table 1.

**Table 1:** Complexities of ${\mathbf{pf}}$ operators

Operator	Worst case	Expected case
${\mathbf{pf}}_\text {sort}$	$O(N \log N)$	$O(N \log N)$
${\mathbf{pf}}_\text {isort}$	$O(N^2)$	$O(N \log N)$
${\mathbf{pf}}_\text {lex}$	*O*(*N*)	*O*(*N*)
${\mathbf{pf}}_\text {smooth}$	$O(N^2)$	$O(N \log N)$
${\mathbf {pf}}_\text {nosort}$	$O(N^2)$	$O(N \log N)$

The operator ${\mathbf{pf}}_\text {lex}$ has the best complexity in both worst and expected case, but it also makes the strongest assumptions. In the expected case, ${\mathbf{pf}}_\text {isort}, {\mathbf{pf}}_\text {smooth},$ and even ${\mathbf{pf}} _\text {nosort}$ asymptotically catch up with ${\mathbf{pf}} _\text {sort}$, whose separate $O(N \log N)$ sorting phase gets no benefit from the elimination of dominated elements.

In a dynamic programming approach, the $\mathbf pf$ operation is executed in the innermost loop of the program, and therefore, constant factors are also relevant. In particular, ${\mathbf{pf}} _\text {nosort}$ becomes interesting as it makes the weakest assumption by not requiring lists to be sorted at any time, in contrast to ${\mathbf{pf}} _\text {lex}$. We will return to this aspect with our applications.

### Pareto merge in linear time

We now specify an implementation of the Pareto merge operation $\mathop {\vee }\limits ^{p}$ which makes use of the fact that its arguments are Pareto sets, represented as lists in decreasing order by the first component (and in increasing order by the second).

$$\begin{aligned} \begin{array}{ll} \mathrm{Input{:}} &{} \mathrm{two \, sorted \, Pareto \, lists.} \\ \mathrm{Output{:}} &{} \mathrm{a \, sorted \, Pareto \, list.} \end{array} \end{aligned}$$

$$\begin{aligned} \begin{array}{lcl} [~] \mathop {\vee }\limits ^{p}y &{} \rightarrow &{} y \\ x \mathop {\vee }\limits ^{p}[~] &{} \rightarrow &{} x \\ (a,b):x \mathop {\vee }\limits ^{p}(c,d):y &{} \rightarrow &{} {{\mathbf {\mathbf{{ case~ }}}}} (a,b) ? (c,d) {{\mathbf {\mathbf{{~of}}}}} \\ {{\mathbf {\mathbf{{case~ }}}}} (>,>) &{} {:} &{} (a,b):(x \mathop {\vee }\limits ^{p}(\text {dropWhile} (\lambda (u,v). v \le b), y)) \\ {{\mathbf {\mathbf{{case~ }}}}} (>,=) &{} {:} &{} (a,b):(x \mathop {\vee }\limits ^{p}y) \\ {{\mathbf {\mathbf{{case~ }}}}} (>,<) &{} {:} &{} (a,b):(x \mathop {\vee }\limits ^{p}((c,d):y)) \\ {{\mathbf {\mathbf{{case~ }}}}} (=,>) &{} {:} &{} (a,b):(x \mathop {\vee }\limits ^{p}(\text {dropWhile} (\lambda (u,v). v \le b), y)) \\ {{\mathbf {\mathbf{{case~ }}}}} (=,=) &{} {:} &{} (a,b):(x \mathop {\vee }\limits ^{p}y) \\ {{\mathbf {\mathbf{{case~ }}}}} (=,<) &{} {:} &{} (c,d):((\text {dropWhile} (\lambda (u,v). v \le d), x) \mathop {\vee }\limits ^{p}y) \\ {{\mathbf {\mathbf{{case~ }}}}} (<,>) &{} {:} &{} (c,d):((a,b):x \mathop {\vee }\limits ^{p}y) \\ {{\mathbf {\mathbf{{case~ }}}}} (<,=) &{} {:} &{} (c,d):(x \mathop {\vee }\limits ^{p}y) \\ {{\mathbf {\mathbf{{case~ }}}}} (<,<) &{} {:} &{} (c,d):((\text {dropWhile} (\lambda (u,v). v \le d), x) \mathop {\vee }\limits ^{p}y ) \end{array} \end{aligned}$$

The function $\text {dropWhile}(p,l)$ walks down a list *l* until it finds an element that does not satisfy the predicate *p*. It returns this element and the remaining list. We use it to eliminate elements smaller than *b* (resp. *d*) in the second dimension. At first glance, the combination of $\mathop {\vee }\limits ^{p}$ and $\text {dropWhile}$ reminds of an $O(N^2)$ algorithm, but this is not true. For input lists of length $n_1$ and $n_2$, where $N = n_1+n_2$, the output list has at most length *N*. It requires at most *O*(*N*) calls to $\mathop {\vee }\limits ^{p}$. $\text {dropWhile}$ requires $k+1$ calls when it deletes *k* elements, with $k \in O(N)$. However, each element deleted by $\text {dropWhile}$ safes a subsequent call to $\mathop {\vee }\limits ^{p}$. Overall, the number of steps remains within *O*(*N*).

### Pareto set extension

Dynamic programming is governed by Bellman’s principle of optimality, which the objective function $\psi = \varphi \circ \sigma$ must obey. Choice must distribute over scoring, which is computed incrementally from smaller to larger sub-solutions. The score $\sigma$ is computed by a combination of local scoring functions $\{f\}$. In Pareto optimization, *f* takes for form $f((a,b)) = (f_\text {A}(a),f_\text {B}(b))$. For each such function *f*,

13 $$\begin{aligned} \varphi (\{f(x),f(y)\}) = f(\varphi (\{x,y\})) \end{aligned}$$

must hold. (The above equation is formulated here for the simplest case: a unary function *f* and a choice function that returns a singleton result). This requirement implies the functions *f* to be *strictly monotone* in each argument that is a subproblem result. (The score functions may take other arguments, too, which are taken from the problem instance).

By *Pareto set extension* we mean the computation of $f(X) = \{f(x) | x \in X\}$, $f(X,Y) = \{f(x,y) | x \in X, y \in Y\}$, and so on for more arguments. On the partially ordered set $(A \times B, \succ )$, we call *f* strictly monotone if $f_\text {A}$ and $f_\text {B}$ are strictly monotone on *A* and *B*, respectively.

#### **Lemma 2.1**

*Pareto set extension. The extension of a Pareto set under a strictly monotone, unary functionfis a Pareto set*.

#### *Proof*

We must show that *f*(*X*) holds no dominated elements. Assume *f*(*X*) holds a dominated element $f((a',b'))$, dominated by some *f*((*a*, *b*)). We have $f_\text {A}(a) >_\text {A} f_\text {A}(a')$ or $f_\text {B}(b) >_\text {B} f_\text {B}(b')$. Strict monotonicity implies $a >_\text {A} a'$ or $b >_\text {B} b'$, implying $(a,b) \succ (a',b')$ in contradiction to the prerequisite that *X* is a Pareto set. $\Box$

The same reasoning does not apply for functions *f* with multiple arguments. Let $f_\text {A} = f_\text {B} = (+)$. We have $f(\{(4,1),(3,2)\}, \{(3,3), (1,4)\}) = \{(7,4),(5,5),(6,5),(4,6)\},$ where $(6,5) \succ (5,5)$ and this extension is not a Pareto set.

Dressing up Pareto optimization for dynamic programming, we must (i) formulate conditions under which $\psi _1\,\, {*}_{\text {Par}}\,\,\psi _2$ fulfills Bellman’s principle, and (ii) show how the Pareto front of the overall solution can be computed incrementally and efficiently from Pareto fronts of sub-solutions, using a combination of the techniques introduced above. Heretofore, these issues had to be resolved with every dynamic programming algorithm that uses Pareto optimization, such as the one by Sitarz or Schnattinger et al. [19, 20]. Striving for general results for a whole class of algorithms, we resort to the framework of ADP. $\square$

## Pareto optimization in ADP

Algebraic dynamic programming (ADP) is a framework for dynamic programming over sequential data. Its declarative specifications achieve a perfect separation of the issues of search space construction, tabulation, and scoring, in clear contrast to the traditional formulation of dynamic programming algorithms by matrix recurrences. Therefore, ADP lends itself to the investigation of Pareto optimization in dynamic programming in general, i.e. independent of a particular DP algorithm. The base reference on ADP is [23] and the lexicographic product was introduced in [10]. ADP in practice is supported by implementations of the framework embedded in Haskell [27] or as an independent domain-specific language and compiler in the Bellman’s GAP system [28, 29]. The results in the present article suggest to extend these systems by a generic Pareto product on evaluation algebras, i.e. to provide the operator ${*}_{\text {Par}}$ as a language feature.

In this section, we recall the basic definitions of ADP (Signatures, evaluation algebras, and tree grammars), in order to relate the Pareto product to other product operators (Relation between Pareto and other products) and prove our main theorem (Preservation of Bellman's principle by the Pareto product). We also show a hand-crafted case of a Pareto product, and its reformulation in ADP (A hand-crafted use case of the Pareto product).

### Algebraic framework

#### Signatures, evaluation algebras, and tree grammars

Let $\mathcal {A}$ be an alphabet and $\mathcal {A}^*$ the set of finite strings over $\mathcal {A}$. A *signature*$\Sigma$ is a set of function symbols and a data type place holder (also called a sort) *S*. The return type of an $f \in \Sigma$ is *S*, each argument is of type *S* or $\mathcal {A}$. $T_{\Sigma }$ denotes a term language described by the signature $\Sigma$ and $T_{\Sigma }(V)$ is the term language with variables from the set *V*. A $\Sigma$*-algebra* or *interpretationA* is a mathematical structure given by a carrier set $S_\text {A}$ for *S* and functions $f_A$ operating on this set for each $f \in \Sigma$, consistent with their specific type. Interpreting $t \in T_{\Sigma }$ by *A* is denoted *A*(*t*) and yields a value in $S_\text {A}$. An *evaluation algebraA* is a $\Sigma$-algebra augmented with an *objective function*$\varphi _\text {A}:[S]\rightarrow [S]$, where square brackets denote multisets.

A *regular tree grammar*$\mathcal {G}$ over a signature $\Sigma$ is defined as tuple $(V,\mathcal {A},Z,P)$, where *V* is the set of non-terminal symbols, $\mathcal {A}$ is an alphabet, *Z* is the axiom and *P* a set of production rules. Each production is of form

14 $$\begin{aligned} v \rightarrow t \text { with } v\in V, t \in T_{\Sigma }(V). \end{aligned}$$

The *regular tree language*$L(\mathcal {G})$ of $\mathcal {G}$ is the subset of $T_{\Sigma }$ that can be derived from *Z* by the rules in *P*.

#### ADP semantics

For input sequence *z*, the tree grammar $\mathcal {G}$ defines the *search space* of the problem instance

15 $$\begin{aligned} X_z = \{x \in L(\mathcal {G}) ~|~ yield(x) = z\}, \end{aligned}$$

where *yield* is the function returning the alphabet symbols decorating the leaves of the tree. (For our present purpose, the reader needs not worry about technical details and can take tree grammars as black-box generators of the search space).

Given a tree grammar $\mathcal {G}$, an evaluation algebra *A* with choice function $\varphi _\text {A}$, and input sequence *z*, an ADP problem is solved by computing

16 $$\begin{aligned} \mathcal {G}(A,x) := \varphi _A([A(x) ~|~x \in X_z]) \end{aligned}$$

While this declarative formulation suggests a three-phase computation—construct *X*, evaluate to *A*(*X*), choose from it via $\varphi _A$—an ADP compiler adds in the amalgamation of these three phases, as it is typical for dynamic programming. For this dynamic programming machinery to work correctly, the algebra *A* must satisfy Bellman’s principle of optimality, stated in full generality [30] by the requirements

17 $$\begin{aligned} \varphi _A [f_A (x_1, \ldots , x_k)|x_1 \leftarrow X_1, \ldots , x_k \leftarrow X_k]= \varphi _A [f_A (x_1, \ldots , x_k)|x_1 \leftarrow \varphi _A(X_1), \ldots , x_k \leftarrow \varphi _A(X_k)] \end{aligned}$$

18 $$\begin{aligned} \varphi _A [X_1 \cup X_2]=\varphi _\text {A} (\varphi _\text {A} (X_1) \cup \varphi _A (X_2))\end{aligned}$$

19 $$\begin{aligned} \varphi _A []=[] \end{aligned}$$

where the $X_i$ denote multisets (reflecting that the same intermediate result can be found several times; this is why we write $x \leftarrow X$ instead of $x \in X$) and $f_A$ is any function from the underlying signature. Note that for nullary functions $f_\text {A}$ (constants) (17) is trivially satisfied as it simplifies to the identity

20 $$\begin{aligned} \varphi _\text {A}([f_\text {A}]) = \varphi _\text {A}([f_\text {A}]) . \end{aligned}$$

When the choice function maximizes or minimizes over a total order, Bellman’s principle implies the strict monotonicity of the scoring functions (Lemma 3.2 below). When only some maximal or minimal solution is sought, one could relax this condition to weak monotonicity, but when all optimal solutions, or even near-optimals are desired, monotonicity must be strict [31]. The formulation given above is more general than the monotonicity requirement, as it also applies to arbitrary objective functions where there may be no maximization or minimization involved, such as candidates counting or enumeration.

#### Products of algebras

Combinations of multiple optimization objectives can be expressed in ADP by *products of algebras*. For all variants of the product operator $(*)$, we define

21 $$\begin{aligned} f_\text {A * B}((a_1,b_1), ..., (a_m,b_m)) = (f_\text {A}(a_1,...,a_m), f_\text {B}(b1, ...,b_m)) \end{aligned}$$

These functions compute independently scores in the Cartesian product of *A* and *B*. By contrast, objective functions are combined in different ways by different product operators.

The lexicographic product, for example, is an evaluation algebra over $\Sigma$ and the objective function of $A {*}_{\text {lex}}B$ is:

$$\begin{aligned} \varphi _{A {*}_{\text {lex}}B}[(a_1,b_1), \ldots ,(a_m,b_m)] =[(l,r)]| l \in set(\varphi _\text {A}[a_{1}, \ldots , a_\text {m}]), r \leftarrow \varphi _{B}[r'|(l',r') \leftarrow [(a_1,b_1), \ldots , (a_\text {m},b_\text {m})], l'=l] \end{aligned}$$

In this formula, *set*(*X*) reduces the multiset *X* to a set. So, $A {*}_{\text {lex}}B$ implements the lexicographical ordering of the two independent criteria as its objective. Aside from ${*}_{\text {lex}}$, Bellman’s GAP also implements a Cartesian and (in a restricted form) a so-called “interleaved” product. To our knowledge, a Pareto product operator has not yet been considered for inclusion in ADP compilers.

### Relation between Pareto and other products

As we show next, Pareto optimization can rightfully be considered as the most general of the combinations discussed here. This holds strictly in the sense that from the Pareto front, the solutions according to the other combinations can be extracted.

#### **Theorem 3.1**

(Pareto front subsumption)* For any grammar*$\mathcal {G}$*, scoring algebrasA andB satisfying Bellman’s principle, and input sequencex, consider the algebra combinations*$A\,\, {*}_{\text {+}\lambda }\,\,B$*, *$A\,\, {*}_{\text {lex}}\,\,B$*, and*$A\,\, {*}_{\text {Par}}\,\,B$.

1. $$\mathcal {G}(A\,\, {*}_{\text {+}\lambda }\,\,B,x) = (\varphi _A\,\, {*}_{\text {+}\,\,\lambda }\varphi _B)(\mathcal {G}(A \,\,{*}_{\text {Par}}\,\,B, x))$$
2. $$\mathcal {G}(A\,\, {*}_{\text {lex}}\,\,B,x) = (\varphi _A\,\, {*}_{\text {lex}}\,\,\varphi _B)(\mathcal {G}(A\,\, {*}_{\text {Par}}\,\,B, x))$$

#### *Proof*

1. Let *t* be the optimal candidate chosen by the left-hand side. Its score is $\lambda A(t) + (1-\lambda ) B(t)$, by (7) and by the definition of (${*}_{\text {+}\lambda }$). This score is maximal. Hence, candidate *t* must be in the Pareto front computed on the right-hand side, represented by the Pareto-optimal pair (*A*(*t*), *B*(*t*)). It could only be missing in the Pareto front if there was another candidate $t'$ dominating *t*, i.e. with $A(t')>A(t)$ and $B(t') \ge B(t)$ or vice versa. But then, we would have $\lambda A(t') + (1-\lambda ) B(t') > \lambda A(t) + (1-\lambda ) B(t)$, contradicting the optimality of *t*. Conversely, no candidate $t''$ in the Pareto front can score strictly higher that *t*, because then, this candidate would have been returned instead of *t*.
2. The same reasoning applies to the lexicographic combination.

The above argument is formulated for an application of the choice function to a complete search space of candidates. By virtue of Bellman’s principle satisfied by *A*, *B*, and their products, the argument inductively holds (by structural induction on the candidates involved) when the choice functions are applied at intermediate steps during the dynamic programming computation. That $A\,\, {*}_{\text {Par}}\,\,B$ also satisfies Bellman’s principle will be shown in our main theorem. $\square$

### A hand-crafted use case of the Pareto product

Any dynamic programmer can hand-craft a Pareto product $A {*}_{\text {Par}}B$ from two evaluation algebras. This requires a substantial programming and debugging effort. Our intention is that this human effort can avoided by a *general* technique delegated to an ADP compiler. This idea was inspired by the work of Schnattinger et al.  [20, 21]. Their algorithm computes via dynamic programming the Pareto front for the “Sankoff problem” of joint RNA sequence alignment and consensus structure prediction [3]. This is their algorithm *in facsimile*:

22 $$\begin{aligned} S(i,j,k,l) \; = \; \text { Pareto-Max } \nonumber \\ \; \left\{ \begin{array}{lll} \; s + \overrightarrow{\gamma } \; : \; s\in S(i,j-1,k,l) \nonumber \\ \cup \; s + \overrightarrow{\gamma } \; : \; s\in S(i,j,k,l-1) \nonumber \\ \cup \; s + \overrightarrow{\sigma } (X_j, Y_l) \; : \; s\in S(i,j-1,k,l-1) \nonumber \\ \underset{h,q}{\bigcup } \; \left\{ \begin{array}{l} s + d \; \nonumber \\ s \in S(i,h-1,k,q-1) \; \nonumber \\ d \in D(h,j,k,l) \; \nonumber \\ \end{array} \right. \; \; \end{array} \right. \; \nonumber \\ D(i,j,k,l) \; = \; s + \overrightarrow{\Psi _{i,j}^{X}}+\overrightarrow{\Psi _{k,l}^{Y}} \; : \; s \in S(i,j-1,k,l-1) \\ S(i,i,k,l) \; = \; \overrightarrow{\gamma }(l-k) \; : \; l>k \nonumber \\ S(i,j,k,k) \; = \; \overrightarrow{\gamma }(j-i) \; : \; j>i \nonumber \\ S(i,i,k,k) \; = \; \overrightarrow{0} \nonumber \end{aligned}$$

Here, $\overrightarrow{\Psi _\text {i,j}^{X}}$ is the probability that *i* and *j* be paired in the sequence *X*. This is computed independently of the alignment score, which is composed of $\overrightarrow{\gamma }$, the gap penalty and $\overrightarrow{\sigma }(X_\text {j}, Y_\text {l})$, the alignment score between the* j*th base in *X* and the* l*th base in *Y*.

The authors demonstrated their algorithm respects Bellman’s principle and correctly computes the Pareto front of its search space. The proof essentially uses the fact that both scores are additive. They showed that all Pareto solutions are generated by the algorithm and that no Pareto solutions are lost during the computation. They based their proof on monotonicity in order to show that the three first terms of the computation of *S*(*i*, *j*, *k*, *l*) consist in summing constant vectors to the current solutions, which leads to the conservation of the dominant solutions. They also showed that the last term needs only dominant solutions contribute to the final result. So, previous deletions of dominated solutions do not lead to a loss of Pareto overall optima. However, in this problem formulation, the general nature of the proof is not easily recognized. We now reformulate this algorithm in the algebraic framework. The correctness of the Pareto optimization then follows from our main theorem below.

#### Sankoff problem signature and algebras

The signature and algebras used to compute base pair probabilities (PROB) and similarity between two sequences (SIM) are presented in Table 2.

**Table 2:** Two evaluation algebras for the Sankoff problem

**SIGNATURE**		**PROB**	**SIM**	
*nil*	$=$	0	0	
*NoStr *(*x*, *y*)	$=$	*x* + *y*	*x* + *y*	
*Split *(*x*, *y*)	$=$	*x* + *y*	*x* + *y*	
*Pair * $(\langle a, b\rangle , x, \langle c, d\rangle )$	$=$	*x* + $\Psi (a, c)$ + $\Psi (b, d)$	*x* + $\sigma (a,b)$ + $\sigma (c,d)$	(*)
*Ins * $(\langle \epsilon , b\rangle )$	$=$	0	$\gamma (b)$	
*Del * $(\langle a, \epsilon \rangle )$	$=$	0	$\gamma (a)$	
*Match * $(\langle a, b \rangle )$	$=$	0	$\sigma (a,b)$	
$\varphi$	$=$	Max	Mx	

The function $\Psi (x, y)$ returns the probability that the bases *x* and *y* be paired. These base pair probabilities are computed as a preliminary step. The functions $\sigma (x, y)$ returns 1 if $a=b$ and else, it returns 0. The function $\gamma ()$ returns the penalties for insertion or deletion, here it is −3. The line marked (*) corresponds to the Eq. 22 of the original algorithm.

#### Tree grammar $\mathcal {G}_{{Sankoff}}$ for the Sankoff problem

For the Sankoff problem, there are two input sequences, refered to in the form $\langle x,y \rangle$.

#### Calling the Sankoff program

Calling either $\mathcal {G}_{{Sankoff}}(SIM, \langle x,y \rangle )$ or $\mathcal {G}_{{Sankoff}}(\text {PROB}, \langle x,y \rangle )$, we either align for maximal similarity, or for maximal base pairing. To solve the problem with Pareto optimization, we call

$$\begin{aligned} \mathcal {G}_{{Sankoff}}(\text {SIM} {*}_{\text {Par}}\text {PROB}, \langle x,y \rangle ). \end{aligned}$$

### Preservation of Bellman’s principle by the Pareto product

In this section we present our main theorem, showing that the Pareto product *always* preserves Bellman’s principle. For the Pareto product to apply, we have the prerequisite that algebras *A* and *B* both maximize over a total order. In this situation, Bellman’s principle specializes as follow:

#### **Lemma 3.2**

*If*$\varphi$* maximizes over a total order, Eq.* (17)* implies that allk-ary functionsf for*$k>0$* are strictly monotone with respect to each argument*.

#### *Proof*

Assume Eq. (17) holds and max stands for $\varphi$. If strict monotonicity was violated, there would be a value pair such that $x > y$, but $f(...,x,...) \le f(...,y,...)$, with all other arguments of *f* unchanged. Then $max[f(max[x,y])] = [f(x)]$, whereas *max*[*f*(*x*), *f*(*y*)] is either [*f*(*x*), *f*(*y*)] if both are equal, or otherwise it is [*f*(*y*)]. In either case, (17) is violated. $\square$

#### **Theorem 3.3**

*The Pareto product preserves Bellman’s principle*.

#### *Proof*

Under the premise that algebras *A* and *B* satisfy Bellman’s principle of optimality, we must show that $A {*}_{\text {Par}}{} B$ satisfies Eqs. (17–19). The algebra functions in the product algebra are $f_\text {A * B}$, cf. (21), and the choice function is ${\mathbf{pf}} _{>_\text {A},>_\text {B}}$.

${\mathbf{pf}} _{>_\text {A},>_\text {B}}$ satisfies (19). This is a trivial consequence of Eq. (6).

${\mathbf{pf}} _{>_\text {A},>_\text {B}}$ satisfies (18). We have to show that

$$\begin{aligned} {\mathbf{pf}} (X \cup Y)={\mathbf{pf}} ({\mathbf{pf}} (X) \cup {\mathbf{pf}} (Y)) \end{aligned}$$

where ${\mathbf{pf}}$ is short for ${\mathbf{pf}} _{>_\text {A},>_\text {B}}$. W.l.o.g assume $x\in X$. The element $x \in X \cup Y$ is in ${\mathbf{pf}} (X \cup Y)$ if and only if it is not dominated by any other element in $X \cup Y$. This implies $x \in {\mathbf{pf}} (X)$, and *x* is not dominated by an element in ${\mathbf{pf}} (Y) \subseteq Y$. Hence, $x \in {\mathbf{pf}} ({\mathbf{pf}} (X) \cup {\mathbf{pf}} (Y))$. Conversely, $x \in X \cup Y$ is not in ${\mathbf{pf}} (X \cup Y)$ if and only if it is dominated by some element $z \in X \cup Y$. Because of transitivity of $\succ$, it will be also dominated by a *dominant* element in *X* or *Y*, which is a member of ${\mathbf{pf}} (X)$ or ${\mathbf{pf}} (Y)$, respectively. Hence, *x* is not in ${\mathbf{pf}} ({\mathbf{pf}} (X) \cup {\mathbf{pf}} (Y))$.

${\mathbf{pf}} _{>_\text {A},>_\text {B}}$ satisfies (17). If $f_{\text {A} {*}_{\text {Par}}\text {b}}$ is a constant (nullary) function, it satisfies (17) because of (20).

For the other functions, we have to show that

$$\begin{aligned}&{\mathbf{pf}} ([f_\text {A * B} (x_1, \ldots , x_\text {k})|x_1 \leftarrow X_1, \ldots , x_\text {k} \leftarrow X_\text {k}])=\\&{\mathbf{pf}} ([f_\text {A * B}(x_1, \ldots , x_\text {k})|x_1 \leftarrow {\mathbf{pf}} (X_1), \ldots , x_\text {k} \leftarrow {\mathbf{pf}} (X_\text {k})]) \end{aligned}$$

with ${\mathbf{pf}}$ short for ${\mathbf{pf}} _{>_\text {A},>_\text {B}}$. It is clear that the right-hand side is a subset of the left-hand side, so we only have to show that no dominating elements are lost. From Lemma 3.2 we know that $f_\text {A}$ and $f_\text {B}$ are strictly monotone in each argument position. Now consider $f_\text {A * B}(...,(a_\text {i},b_\text {i}),...)$. With all other arguments equal, $f_\text {A}(...,a_\text {i},...) \succ f_\text {A}(...,a_\text {i'},...)$ if and only if $a_\text {i }\succ a_\text {i'}$, and the same for $f_\text {B}$. We conclude that $f_\text {A * B}(...,(a_\text {i},b_\text {i}),...) \succ f_\text {A * B}(...,(a_\text {i'},b_\text {i'}),...)$ if and only if $(a_\text {i},b_\text {i}) \succ (a_\text {i'},b_\text {i'})$, and hence $f_\text {A * B}$ is strictly monotone with respect to the partial ordering $\succ$. If $(a_\text {i},b_\text {i}) \succ (a_\text {i'},b_\text {i'})$ in $X_\text {i}$ and hence $(a_\text {i'},b_\text {i'}) \notin {\mathbf{pf}} (X_\text {i})$, then the element $f_\text {A * B}(...,(a_\text {i'},b_\text {i}),...)$ will not be considered on the left-hand side. But anyway, it would be dominated by $f_\text {A * B}(...,(a_\text {i},b_\text {i}),...)$ and could not enter the overall result. $\square$

While our theorem guarantees that $(A {*}_{\text {Par}}B)$ satisfies Bellman’s principle under the above prerequisites, an ADP compiler providing the ${*}_{\text {Par}}$ operation on evaluation algebras cannot check these prerequisites. In general, it cannot prove that $\varphi _A$ and $\varphi _B$ maximize over a total order, nor can it ensure strict monotonicity. However, there may be obvious abuses of ${*}_{\text {Par}}$ that a compiler can safeguard against.

## Implementation

The Pareto product can be implemented simply by providing the Pareto front operator $\varphi = {\mathbf{pf}} _{>_\text {A,}>_\text {B}}$ as the choice function for the algebra product $(A {*}_{\text {Par}}{} B)$. In this case, the results of ${\mathbf{pf}} _{>_\text {A,}>_\text {B}}$ can be represented as sorted or unsorted lists. A more ambitious implementation would monitor the status of intermediate results as lexicographically sorted lists, to take advantage of the more efficient Pareto front operator ${\mathbf{pf}} _\text {lex}$ or ${\mathbf{pf}} _\text {smooth}$ on sorted lists.

We will describe these implementation options by means of an example production which covers the relevant cases. A tree grammar describing an ADP algorithm has an arbitrary number of productions, but their meaning is independent.

Let *f*, *g*, and *h* be a binary, an unary and a nullary scoring function from the underlying signature. A tree grammar rule such as specifies the computation of partial results for a subproblem of type *W* from partial results already computed from subproblems of types *X* and *Y*, of type *Z*, or for an empty subproblem via a (constant) scoring function *h*. It is important to have a binary operation in our example, as this type of Pareto set extension does not perserve the Pareto property, and hence is a more difficult case (cf. Lemma 2.1). Beyond this, signature functions may have arbitrary arity, and trees on the right-hand side can have arbitrary height. These cases can be handled in analogy to what we do next.

We use the nonterminal symbols also as names for the list of subproblem solutions derived from them. Hence, we compute a list of answers $W = [w_1,w_2,...]$ from $X = [x_1, x_2,...]$ and so on. Note that *h* denotes a constant list, in most cases a singleton, but not necessarily so. We do not have to worry about indexing subproblems and dynamic programming tables, as this is added by the standard ADP machinery.

### Standard implementation

Candidate lists are created by terminal grammar rules, by extension of intermediate results with scoring functions, and by union of answers from alternative rules for the same nonterminal.

We describe the standard implementation by three operators $\mathbf{\otimes }, {\oplus }, \mathbf{~\#~ }$, respectively pronounced “extend”, “combine” and “select”.

Let $C = (A \times B)$, and let $\mathcal {L}$ denote the powerset of *C*. Elements of $\mathcal {L}$ are simply lists over *C* in our implementation. $(\mathcal {L} \rightarrow \mathcal {L})$ denotes functions over subsets of *C*, such as our choice functions. Our operators have the following types:

23 $$\begin{aligned} \mathbf{~\#~ }:\,&\mathcal {L} \times (\mathcal {L} \rightarrow {S})&\rightarrow \mathcal {L}\end{aligned}$$

24 $$\begin{aligned} {\oplus }~ :&\,\mathcal {L} \times \mathcal {L}&\rightarrow \mathcal {L}\end{aligned}$$

25 $$\begin{aligned} \mathbf{\otimes }~ :&\,\mathcal {L} \times (C \rightarrow C)&\rightarrow \mathcal {L}\end{aligned}$$

26 $$\begin{aligned} \mathbf{\otimes }~ :&\,\mathcal {L} \times \mathcal {L} \times (C \times C \rightarrow C)&\rightarrow \mathcal {L} \end{aligned}$$

The type of $\mathbf{\otimes }$ is overloaded according to the arity of its function argument, which is arbitrary in general. For our exposition, we need only arities 1 and 2. (This flexible arity overloading explains why we do not use infix notion with $\mathbf{\otimes }$.) The operators are defined as follows:

27 $$\begin{aligned} l \mathbf{~\#~ }{\mathbf{pf}}= {\mathbf{pf}} (l) \end{aligned}$$

28 $$\begin{aligned} l_1 {\oplus }~l_2= l_1 \text{++ } \,l_2 \end{aligned}$$

29 $$\begin{aligned} \mathbf{\otimes }(f,X,Y)= [f(x,y) ~|~ x \in X, y \in Y] \end{aligned}$$

30 $$\begin{aligned} \mathbf{\otimes }(g,X)= [g(x) ~|~ x \in X] \end{aligned}$$

Operator $\mathbf{~\#~ }$ simply applies the choice function to a list *l* of intermediate results (27), generally the function $\varphi$, and ${\mathbf{pf}}$ in our specific case. We append lists of solutions with ${\oplus }$ (28), and $\mathbf{\otimes }$ extends solutions from smaller subproblems to bigger ones (29, 30). Note that there is no requirement on the constant scoring function *h*. Typically, such a function generates an empty list or a single element anyway. In general however, it may produce a list of alternative answers, and this need not be a Pareto list in the standard implementation.

Using this set of definitions, our example production describes the computation of

$$\begin{aligned} W = (\mathbf{\otimes }(f,X,Y) ~~{\oplus }~~ \mathbf{\otimes }(g,Z)) ~~{\oplus }~~ h) \mathbf{~\#~ }{\mathbf{pf}} . \end{aligned}$$

Any of our variants ${\mathbf{pf}} _\text {sort},{\mathbf{pf}} _\text {isort}, {\mathbf{pf}} _\text {nosort}$ can be used for ${\mathbf{pf}}$, but not the linear-time ${\mathbf{pf}} _\text {lex}$, because in (28) and (29), lists come out unsorted.

### Lexicographically sorted implementation

This implementation defines the operators ${\oplus }$ and $\mathbf{\otimes }$ such that they keep intermediate lists sorted. As a consequence, the Pareto front operator ${\mathbf{pf}}$ can be replaced by the more efficient ${\mathbf{pf}} _\text {lex}$.

31 $$\begin{aligned} l \mathbf{~\#~ }{\mathbf{pf}}= {\mathbf{pf}} _\text {lex}(l) \end{aligned}$$

32 $$\begin{aligned} l_1 {\oplus }~l_2= merge(l_1, l_2) \end{aligned}$$

33 $$\begin{aligned} \mathbf{\otimes }(f,X,Y)= foldrmerge( [[f(x,y)~ |~ x \leftarrow X]~|~ y \in Y]) \end{aligned}$$

34 $$\begin{aligned} \mathbf{\otimes }(g,X)= [g(x)~|~ x \leftarrow X] \end{aligned}$$

35 $$\begin{aligned} h= sort(h) \end{aligned}$$

The function *merge* merges two sorted lists in linear time, such that the result is sorted, and *foldrmerge* does so iteratively for a list of sorted lists.

We show by structural induction that all intermediate solution lists are sorted. Eq. (35) covers the base case. It requires that a constant function such as *h* produces its answer list in sorted form. Now consider the recursive cases, assuming that lists *X* and *Y* are sorted. In Eq. (34), the constructed list is sorted. This follows from the prerequisite that algebras *A* and *B* satisfy Bellman’s principle, and hence *g* is monotonic in *both* algebras. Thus, given a sorted list *X*, the list $[g(x) | x \in X]$ is also sorted lexicographically (Lemma 2.5). In Eq. (33), the above monotonicity argument holds for each of the lists $[f(x,y) | x \in X]$ for *fixedy*. Lists for different $y \in Y$ are merged, which results in an overall sorted list. This also takes linear time. In Eq. (32), two sorted lists are merged into a sorted list. In Eq. (31), ${\mathbf{pf}} _\text {lex}$ finds a sorted list and reduces it to a Pareto list, which by definition is sorted (cf. 2.2).

### Pareto-eager implementation

The standard implementation applies a Pareto front operator after constructing a list of intermediate results. This list is built and combined from several sublists. By our main theorem, the Pareto front operation distributes over combinations of sublists, so we can integrate the $\mathbf{~\#~ }$ operator into the ${\oplus }$ operator. This has the effect that sizes of intermediate results are reduced as early as possible. We define our operators as follows:

36 $$\begin{aligned} l \mathbf{~\#~ }{\mathbf{pf}}=l \end{aligned}$$

37 $$\begin{aligned} l_1 {\oplus }~l_2= l_1 \mathop {\vee }\limits ^{p}l_2 \end{aligned}$$

38 $$\begin{aligned} \mathbf{\otimes }(f,X,Y)= foldr \mathop {\vee }\limits ^{p}[]~ [[f(x,y) ~|~ x \leftarrow X] ~|~ y \leftarrow Y] \end{aligned}$$

39 $$\begin{aligned} \mathbf{\otimes }(g,X)= [g(x) ~|~ x \leftarrow X] \end{aligned}$$

40 $$\begin{aligned} h= {\mathbf{pf}} (h) \end{aligned}$$

Again we argue by structural induction over the candidates in the search space. As the base case, *h* must produce Pareto lists as initial answers (Eq. 40). The $\mathop {\vee }\limits ^{p}$ operation in Eq. (37) can assume argument lists to be Pareto lists already. In Eq. (39), the new list must be a Pareto list due to the extension Lemma 2.5. In Eq. (38), the same holds for each intermediate list $[f(x,y) | x \in X]$ for each *y*, and we can merge them successively. Finally, the $\mathbf{~\#~ }$ operator skips the computation of the Pareto front, as by induction, all the lists that arise at this point are Pareto lists already.

In "Pareto merge in linear time", we showed that $\mathop {\vee }\limits ^{p}$ can be implemented in *O*(*N*), and therefore, each step in the Pareto-eager implementation takes linear time. This means that Pareto optimization incurs no intrinsic overhead, compared to a single objective which returns a comparable number of results. This is an encouraging insight, but leaves us one aspect to worry about: The size *N* of the Pareto front which is computed from an input sequence of length *n*.

### Runtime impact of Pareto front size

For a typical dynamic programming problem in sequence analysis, an input sequence of length *n* creates an exponential search space of size $O(2^n)$. Still, by tabulation and re-use of intermediate subproblem solutions, dynamic programming manages to solve such a problem in polynomial time, say $O(n^r)$. The value of *r* depends on the nature of the problem, and when encoded in ADP, it is apparent as a property of the grammar which describes the problem decomposition [23]. We have $r = 2$ for simple sequence alignment, $r = 3$ for simple RNA structure prediction, $r = 4$ to $r = 6$ for RNA structures including various classes of pseudoknots, and so on. This all applies when a single, optimal result is returned.

For ADP algorithms returning the *k* best results, complexity must be stated more precisely as $O(n^r k^{r-1})$. As long as *k* is a constant, such as in *k*-best optimization, this does not change the asymptotics. However, computing all answers within *p* percent of the optimal score may well incur exponential growth of *k*. Probabilistic shape analysis of RNA has a runtime of $O(n^3 \alpha ^n)$ with $\alpha \approx 1.1$, because the number of shape classes grows exponentially with sequence length [11, 32].

With Pareto optimization, the size *k* of the answer set is not fixed in beforehand. The size of the Pareto front, for a set of size *N*, is expected to be *H*(*N*) (cf. "Pareto sets and the Pareto front operator"). Using $N \in O(2^n)$ and $H(N) \approx \ln (N)$  [24], we can expect an effective size of the result sets in *O*(*n*). Taking all things together, we can compute the Pareto front for an (algebraic) dynamic programming problem in $O(n^{2r-1})$ expected time, where *n* is input length and *r* reflects the complexity of the search space.

In applications, the size of the Pareto front needs not to follow expectation. We may achieve efficiency of $O(n^r k^{r-1})$ where $k \ll n$. Fortunately, in the application scenario of the next section, we find ourselves in this positive situation.

## Applications

### Evaluation goals

In our applications reported here, we persue a twofold goal. (i) Our foremost goal is to determine whether Pareto optimization is practical in some real-world applications. This includes the assessment of constant factors of alternative implementations of the Pareto front operator ${\mathbf{pf}}$. And (ii), we want to demonstrate that Pareto optimization allows us to draw interesting observations about the relative behaviour of two scoring schemes competing for the same purpose. Our applications are taken from the domain of RNA secondary structure prediction. One is the Sankoff problem of simultaneous alignment and folding of two RNA sequences, introduced already above. The two scoring systems are sequence similarity versus base pair probabilities. Our second application choice is the single sequence structure prediction problem, using two alternative scoring functions. One is MFE, the classical minimum free energy folding approach, based on a thermodynamic nearest-neighbour model with about thousand parameters. The other one is MEA, maximum expected accuracy folding, which is a recent refinement of MFE folding. By this method, the MFE approach is first used to calculate base pair probabilities for the folding space of the given sequence, and in a second phase, the structure is determined which maximises the accumulated base pair probabilities.

Our first hypothesis addresses efficiency issues.

#### **Hypothesis A**

Pareto optimization in a realistic scenario is not more expensive than other approaches calculating a similar amount of alternative answers.

We assess hypothesis A in "Runtime and memory measurements" by computing the trade-off between the different Pareto front implementations described in "Implementation". Emprical Pareto front sizes are reported in "Pareto front size". We use the MFE versus MEA application for all these measurements. An interesting occurence of worst case behaviour for Schnattinger’s variant of the Sankoff algorithm is analyzed in "Anti-correlation and real worst case behaviour".

For a more biologically inspired assessment, we chose the Sankoff problem to test

#### **Hypothesis B**

A small Pareto front is indicative of a strong biological signal of homology.

This assessment is shown in "Pareto solutions in the Sanko algorithm".

We try to get insight into the relationship of the structures that make up the Pareto front, coming back to the application of MFE versus MEA folding.

#### **Hypothesis C**

The Pareto front of MFE versus MEA is comprised of a small number of macrostates, accompanied by essentially the same corona of microstates.

Akin to Pareto optimization, abstract shape analysis of RNA can give us an “interesting” set of alternative foldings [11]. They are characterized by the best-scoring structures having different abstract shapes. This idea being perfectly orthogonal to Pareto optimization, we give some attention to the question how abstract shapes and Pareto optima are related. Here we test the

#### **Hypothesis D**

Abstract shape analysis and Pareto optimization produce about the same set of alternative “interesting” structures.

In the evaluation of hypotheses A–D, our test data for the MFE/MEA application consists of 331 RNA sequences of length 12–356 nucleotides, extracted from the full data set used in [33]. The data set is available with the supplementary material. For the Sankoff problem, we use sequences from two Rfam families. We use $n_1=19$ PreQ1 RNA sequences ($SSTRAND:RF\_00522$) and $n_2=30$ IRE RNA sequences ($SSTRAND:RF\_00037$) extracted from the core data set of the Rfam database [34].

### Algorithms implemented

We give a short sketch of how our algorithms are implemented. For each application, we can re-use grammars and algebras from the RNAshapes repository [35]. It is just the Pareto optimization which is new.

For the standard implementation, we tested the variants ${\mathbf{pf}} _\text {isort}$, ${\mathbf{pf}} _\text {sort}$, ${\mathbf{pf}} _\text {smooth}$, and ${\mathbf{pf}} _\text {nosort}$ (cf. "Computing the Pareto front"). Fortunately, the standard implementation can be mimicked in GAP-L without changing language or compiler^a^. However, we can not evaluate the Pareto-eager implementation based on ${\mathbf{pf}} _\text {lex}$ with Bellman’s GAP, as this would imply extension of GAP-L and modification of the sophisticated code generation in the Bellman’s GAP compiler.

In order to compare the Pareto-eager implementation to the others, we resorted to an implementation of ADP as a Haskell-embedded combinator language [23]. First, we added the variants ${\mathbf{pf}} _\text {isort}$, ${\mathbf{pf}} _\text {nosort}$, and ${\mathbf{pf}} _\text {smooth}$ for the standard implementation. Then, we designed a modified set of combinators, corresponding to the outline in "Pareto-eager implementation". (For the expert: The key idea is to exploit monotonicity and compute the set $\{f(x_\text {i},y_\text {j})\}$ of intermediate results represented as nested lists in the form $[[f(x_\text {i},y_\text {j}) | j = 1,... ] | i = 1,... ]$. For fixed $x_\text {i}$, the sublist $[f(x_\text {i},y_\text {j}) | j = 1,...]$ is sorted if the list $[y_1, ,,,]$ is. This is ensured by structural induction and strict monotonicity of *f* on its second argument position). While this implementation is significantly slower than Bellman’s GAP code, it suffices to compare the Pareto-eager implementation to its alternatives.

We use available building blocks for the independent optimizations: the RNA folding grammar *OverDangle* (avoiding lonely base pairs) and the evaluation algebras *MFE* and *MEA*. In *MFE* and *MEA*, we replace their objective functions by ones that report the *k* best (near-optimal) structures, where *k* is a parameter. This allows us to run *OverDangle*(*MFE*(*k*), *x*) and *OverDangle*(*MEA*(*k*), *x*), with the choice of *k* explained further below.

The size of the folding space *X* for a given sequence *x* is independent of the optimization we perform. The Bellman’s GAP compiler can automatically produce a counting algebra *COUNT*, that determines the size of the folding space. We run *OverDangle*(*COUNT*, *x*) on all our test data to get concrete folding space sizes, to be related to the sizes of their Pareto fronts.

The choice of *k* for a fair evaluation is not obvious. Selecting $k=1$ for *MFE* and *MEA* would be unfair, as the Pareto front provides much deeper information. We considered using $k = E(|X|, |x|=n)$, but the expected size of the search space is not a good predictor, as |*X*| varies strongly with the sequence content of *x*. Therefore, we first run Pareto optimization on *x*, record the size of the Pareto front for this call, and then set *k* to this number when computing *OverDangle*(*MFE*(*k*), *x*) and *OverDangle*(*MEA*(*k*), *x*).

### Runtime and memory measurements

This section and the next are devoted to our

#### **Hypothesis A**

Pareto optimization in a realistic scenario is not more expensive than other approaches calculating a similar amount of alternative answers.

We evaluate the performances of the Pareto front computation, using ${\mathbf{pf}} _\text {isort}(X)$, ${\mathbf{pf}} _\text {sort}(X)$, ${\mathbf{pf}} _\text {smooth}(X)$, and ${\mathbf{pf}} _\text {nosort}(X)$. Note that all compute the same Pareto front, and hence have the same *k* in their asymptotics. For a fair comparison with two single-objective algorithms MFE and MEA, we use their versions *MFE*(*k*) and *MEA*(*k*), computing the *k* best structures under each objective. Here, *k* is set to the actual Pareto front size for the given input (which, of course, is only known because before we also compute the Pareto front with the other algorithms). All programs are compiled by the Bellman’s GAP compiler using the same optimization options [29].

In Table 3 we show computation time and memory consumption, accumulated over all sequences and specifically for the longest sequence. These are our main observations:

**Table 3:** Runtimes and memory requirements for *MFE*(*k*), *MEA*(*k*) (where *k* is the empirical Pareto front size for a given input), and their Pareto product $({MFE}\,\, {*}_{\text {Par}}\,\,{MEA})$, accumulated over 331 sequences (left) and for the longest sequence ($n = 356, k = 38$, right)

Algebra	Time (min)	Memory (GB)	Time (min)	Memory (GB)
*MFE(k)* alone	71	163.68	5	1.16
*MEA(k)* alone	61	153.51	5	1.05
*MFE(k) + MEA(k)*	132 (+)	163.68 (max)	10	1.16 (max)
$$({MFE} {*}_{\text {Par}}{MEA})$$				
$~~~{\mathbf{pf}} _\text {nosort}$	8	197.28	0.22	1.28
$~~~{\mathbf{pf}} _\text {smooth}$	9.5	192.79	0.5	1.28
$~~~{\mathbf{pf}} _\text {sort}$	18	271.21	1	1.28
$~~~{\mathbf{pf}} _\text {isort}$	32	250.21	3	2.11

The computations were performed by using Bellman’s GAP.

1. In terms of runtime, we find that the Pareto optimization performs not only better than the sum of the two independent optimizations, but also better than each of them individually. We attribute this to the fact that the Pareto algorithm adjusts itself to the size of the Pareto front, and this size tends^b^ to be smaller than *k* for small sub-problems. The search space itself, however, is exponentially larger than the Pareto front, and even on small sub-words it provides *k* near-optimals for *MFE*(*k*) and *MEA*(*k*) to spend computation on. This effect is strongest for our longest sequence, where $k = 38$ and the ratio of $({MFE}(k)+{MEA}(k)) / {\mathbf{pf}} _\text {nosort} \approx 45$.
2. The average case behaviour of ${\mathbf{pf}} _\text {nosort}(X)$ is superior to all the sorting implementations of ${\mathbf{pf}}$. This is an unexpected and interesting observation. We attribute this to a positive randomization effect. Comparing a new element to the extremal points of the Pareto front, maximal in one but minimal in the other dimension, is unlikely to establish domination. This what *always happens first* with sorted intermediate lists, and the element will walk along towards the middle of the list until it eventually is found to be dominated. In unsorted lists, a non-extremal element that dominates the new entry will, on average, be encountered earlier.
3. For evaluating the Pareto-eager strategy, we used the Haskell-embedded implementation. In the functional setting, ${\mathbf{pf}} _\text {isort}$ required the least garbage collections and performed best. Somewhat unexpectedly, the eager strategy was consistently a bit slower than ${\mathbf{pf}} _\text {isort}$ and close to ${\mathbf{pf}} _\text {nosort}$, slower only by a factor varying between 1.0 and 1.2. It was faster than ${\mathbf{pf}} _\text {smooth}$, in turn by a factor between 1.1 and 1.5.
4. Memory consumption of Pareto optimization is consistent over different implementations of ${\mathbf{pf}}$. It is higher than either *MFE*(*k*) or *MEA*(*k*) alone, but clearly less than *the sum* of *MFE*(*k*) and *MEA*(*k*). This is better than expected, because after all, it solves both problems simultaneously.

Note that the above values are measurements of constant factors, and averaged over many runs. So, ${\mathbf{pf}} _\text {nosort}$ is not always faster than ${\mathbf{pf}} _\text {smooth}$. In fact, we have seen cases where ${\mathbf{pf}} _\text {nosort}$ is faster than ${\mathbf{pf}} _\text {smooth}$ for ${\mathbf{pf}} _{>_\text {A},>_\text {B}}$, but slower for ${\mathbf{pf}} _{>_\text {A},<_\text {B}}$ (where in the latter case, we switch from maximization to minimization in algebra *B*).

### Pareto front size

The size of the Pareto front is of critical practical importance. Pareto front sizes in the hundreds, even for sequences of moderate length, would be prohibitive. The number of solutions in the Pareto front depends on the data. Not only on the sequence length and the size of the search space in our RNA folding scenario, but also on the actual structures found. For example, if there is a very prominent structure in the folding space, it will dominate many other solutions in *both* objectives, and the Pareto front will be small. On the other hand, in one case we observed a Pareto front of size $\approx |x|$ with the program of Schnattinger et al. on a Sankoff-style algorithm, an effect we will study in detail below.

**Figure 1:**
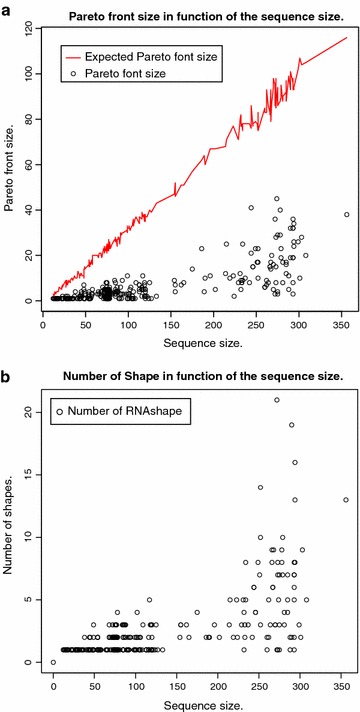
**a** Empirical Pareto front size of ${OverDangle}({MFE} {*}_{\text {Par}}{MEA},x)$ as a function of |*x*|. The red line corresponds the *H*(|*X*|), the expected Pareto front size according to the harmonic law, applied to the empirical value of |*X*| for each *x*.** b** Number of abstract RNA shapes [11] in the Pareto front, in function of the sequence size.

Figure 1 shows our measurements. We observe the following:

- Pareto front sizes are quite moderate, ranging round 10 for $n=100$, 15 for $n = 200$, up to 45 for $n = 274$. Specifically, our longest sequence ($n = 356$) has a Pareto front of size 38.
- Variance is high (as expected), and because of the strong variation, we did not fit a line through our measurement points. However, they are all dominated by the expected size of the Pareto front (red line).
- We did not smooth the graph for *H*(|*X*|), such that it also demonstrates the variance in the search space sizes; just read the y-axis as a logarithmic scale for $e^{H(|X|)}$. The roughly linear behavior conforms with the theoretical analysis.

The moderate sizes of Pareto fronts in our applications also imply that no benefit is to be expected from using quad-tree data structures in place of our sorted list representation. According to measurements in [36], population sizes in the thousands are required to make the more sophisticated data structure pay off.

Summing up our empirical data, we state that Hypothesis A has been confirmed in general, which does not rule out that there are problematic cases. One of these is discussed next.

### Anti-correlation and real worst case behaviour

Two scoring functions are correlated to the extent by which they rank the candidates of the search space in the same order. Perfect correlation or anti-correlation would render a combined application of both objectives meaningless. Perfect positive correlation implies that an optimal candidate under $>_\text {A}$ is also optimal under $>_\text {B}$, so nothing is to be gained from optimizing with respect to $>_\text {B}$. Perfect anti-correlation means that the optimal candidates under $>_\text {B}$ are the worst candidates under $>_\text {A}$. Hence, they can also be obtained as the optimal candidates optimizing under $<_\text {A}$ alone. In interesting scenarios, we can expect the two scoring schemes to correlate in some of the local scoring functions, and anti-correlate in others.

Anti-correlation can make the worst case real, where the size of the Pareto front does not follow the Harmonic law, but is linear the size of the interval of score values actually occurring (cf. Observation 3). There is a minor flaw in the objective function used in [20], harmless at first sight, but provoking worst case behaviour on some inputs. It is instructive to look at the situation in detail.

The objective functions in [20] are adopted from the amalgamated score in [8]. Schnattinger et al. took RNAalifold’s parametrized combination of energy and covariance scoring, dissecting ($\psi _1\,\, {*}_{\text {+}\lambda }\,\,\psi _2)$ literally into ($\psi _1\,\, {*}_{\text {Par}}\,\,\psi _2)$. However, Hofacker et al. had chosen for their combination an engineered variant of similarity scoring, where base pair columns were not scored for sequence similarity (see equation line marked 22). Our algorithm corrects this case, see label $(*)$ in Table 3, making algebra *SIM* a proper similarity score. Literal dissection of this combination in [20] led to two scoring schemes that are negatively correlated in the following case: Choosing a *base pair* increases the covariance score but decreases the sequence similarity score in the case where the individual bases in the paired alignment columns actually match. Amusingly, the worst case occurs when solving the Sankoff problem for two identical sequences! While we would expect a Pareto front of size 1, with no gaps, no mismatches, and a maximal number *p* of base pairs, what we actually get is a worst case Pareto front of *p* elements, because every base pair omitted increases the sequence score.

This observation teaches us that a large Pareto front can result from inadvertent anti-correlation in the scoring functions.

### Pareto solutions in the Sankoff algorithm

We now consider Pareto optimization for the Sankoff problem. The two optimization objectives combined are sequence similarity (*SIM*) and base pair probability of the consensus structure (*PROB*). One can expect both measures to be more correlated when the sequences are in fact closely related and have a conserved consensus structure. We investigate our

#### **Hypothesis B**

 A small Pareto front is indicative of a strong biological signal of homology.

**Figure 2:**
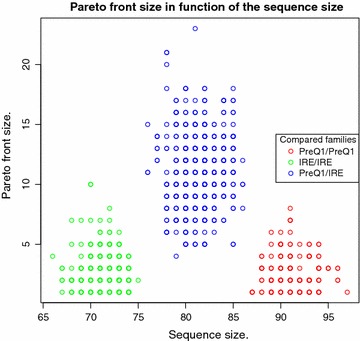
Empirical Pareto front size of $\mathcal {G}_{Sankoff}({PROB} {*}_{\text {Par}}{} {SIM},x)$. The* red plots* correspond to the alignment of two PreQ1 sequences, the* green* ones to the alignment of two IRE sequences and the* blue* ones to the alignment between a IRE sequence and a PreQ1 sequence. Sequence size is the sum of the two aligned sequences size.

A thorough assessment of this hypothesis is outside of the scope of the present article, but we give some evidence that supports it. Our test data consists of $n_1=19$ PreQ1 RNA sequences ($SSTRAND:RF\_00522$) and $n_2=30$ IRE RNA sequences ($SSTRAND:RF\_00037$) extracted from the core data set of the Rfam database [34]. We perform all intra-family and inter-family alignments. The results are shown in Figure 2.

We observe the following:

- The Pareto front size is reduced when comparing sequences from the same family (average Pareto front size of 2.56 for the IRE/IRE and 2.50 for the PreQ1/PreQ1).
- The Pareto front size is larger when aligning two sequences from different families (average Pareto front size of 11). Note that the two families are unrelated, so this can be taken as an experiment on random RNA sequences.
- However, there is some overlap between the extreme cases of both scenarios.

The size of the Pareto front could be useful for deciding family membership, not by itself but as a third criterion in addition to the two scores obtained separately. In fact, one might also be interested in the similarity between the structures that occur in the Pareto front. This is what we consider next.

### Internal structure of the Pareto front of MFE and MEA folding

We now consider each structure in the Pareto front of some sequences extracted from the MFE/MEA dataset with respect to our

#### **Hypothesis C**

 The Pareto front is comprised of a small number of macrostates, accompanied by essentially the same corona of microstates.

By using the RNA movies software [37], we can illustrate the transitions between the different structures in the Pareto front. In the Movie 1, Movie 2, Movie 3 (*cf.* Additional files 1, 2, 3, respectively; these are animated GIFs best viewed with a browser), we can see that there is a single dominating structure (macrostate). The different structures in the Pareto front are all minor modifications of this macrostate. They are local helix modifications or show the formation of small new helices inside big loops. For larger Pareto fronts, we can observe such microstates arranged around several macrostates, indicating very different structures (Additional file 4: Movie 4, Additional file 5: Movie 5, Additional file 6: Movie 6). Each macrostate has a different abstract shape.

Hypothesis C was confirmed in all the cases we studied. This means that one could condense the information in the Pareto front to a small set of macrostate structures, which would have different shapes. This leads us to further explore the relationship between Pareto optimization (based on *MFE* and *MEA*) and abstract shape analysis (based on shape abstraction and *eitherMFE* or *MEA* alone).

### Pareto optimization versus abtract shape analysis

Our Hypothesis D is a somewhat radical statement:

Abstract shape analysis and Pareto optimization produce about the same set of alternative “interesting” structures.

It would make Pareto optimization less attractive in the domain of RNA structure analysis, as well as in other domains where the idea of shape abstraction can be replicated. But our observations refute this hypothesis. Overall, the relation between the two approaches appears to be non-trivial.

**Figure 3:**
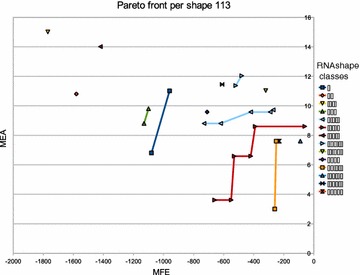
Pareto front per shape for the RNA structure of a hammerhead ribozyme ($SSTRAND:RFA\_00430$).

**Figure 4:**
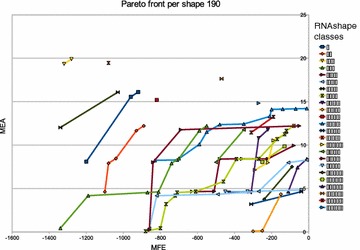
Pareto front per shape for the structures of a tRNA $SSTRAND:SPR\_00243$.

**Figure 5:**
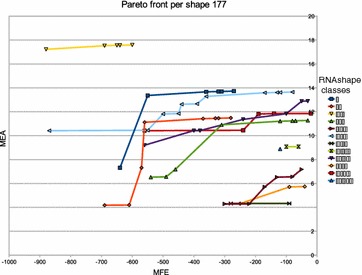
Pareto front per shape for the structures of a tRNA ($SSTRAND:SPR\_00142$).

**Figure 6:**
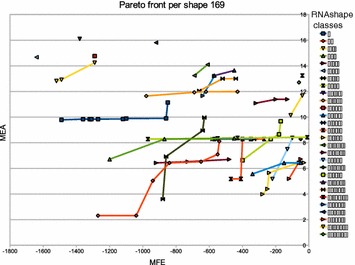
Pareto front per shape for the structures of a tRNA ($SSTRAND:SPR\_00103$).

We checked the ratio of the number of structures in the Pareto front, and the number of different abstract shapes they represent, but this ratio, ranging from 1 to $\approx{12}$, did no exhibit an obvious pattern. Taking a *SHAPE* algebra from the RNAshapes repository, an experiment with ${OverDangle}({SHAPE} * ({MFE} {*}_{\text {Par}}{}\,\, {MEA}), x)$ has been performed. This call computes the Pareto front for each shape. The computation was performed for six sequences extracted from the dataset used for computing the Pareto front between MFE/MEA. The results are presented in the Figures 3, 4, 5 and 6. We see different scenarios occuring:

In Figure 3, we find a dominating shape and a singleton Pareto front. In Figure 4, we find a dominating shape and a Pareto front which holds exactly the *MFE* and *MEA* optima of this shape. In Figure 5, we see a more fine-grained Pareto front with all elements residing in the dominant shape. Figure 6 shows a two-element Pareto front composed of different shapes.

Note that in a case like Figure 4, Pareto optimization with *MFE* and *MEA* will only produce the dominant shape (“[[][]]”, yellow), while in combination with abstract shape analysis, we also see two further macrostates that might be of interest: “[][]” (pink) and “[[][][]]” (dark green). From these observations, we conclude that Hypothesis D is to be refuted. Shape abstraction and Pareto optimization are independent techniques that allow for even deeper analysis in combination.

## Conclusion

Let us review our results, referring back to the questions (i)–(iv) formulated in the introduction. We have shown (i) that the exact Pareto front of two independent objectives can be computed by dynamic programming. The theoretical prerequisite for this is the preservation of Bellman’s principle by the Pareto product operator ${*}_{\text {Par}}$, established in our main theorem.

We have shown (ii) that by the Pareto-eager implementation, one can achieve Pareto optimization without an asymptotic penalty, compared to other optimizations which return a comparable number of results. We have shown (iii) that empirically, for the case of RNA folding under different objectives, the size of the Pareto front remains within moderate bounds, clearly lower than theoretical expectation. All in all, this says the Pareto optimization is practical for sequence analysis and moderate sequence sizes.

We demonstrated (iv) that Pareto optimization allows us to study in depth the relative behaviour of two competing objectives, minimum-free-energy and maximum-expected-accuracy in our application domain. We found in pairwise sequence analysis that, as to be expected, a small Pareto front in the Sankoff problem indicates a potential family relationship.

These findings, of course, create new work items and open questions. Foremost, the implementation of the ${*}_{\text {Par}}$ operator together with its Pareto-eager implementation is up as a challenge to all who work on frameworks supporting dynamic programming in sequence analysis. It will be interesting to see if the Pareto-eager implementation can beat ${\mathbf{pf}} _\text {nosort}$ in terms of constant factors in such implementations.

Many established bioinformatics tools, which so far rely on an ad-hoc combination of different objectives, could be re-evaluated using Pareto optimization. Specifically for RNA structure analysis, in a recent review Rivas argues that in order to further improve predictions, different types of informations must be taken into account [38]. She advocates the conversion of all data sources into a probabilistic framework as a unifying solution. Pareto optimization opens up an alternative route, as it allows to combine multiple objectives without such conversion. This includes the Pareto-style combination of stochastic grammars with other (non-probabilistic) types of information.

Eventually, Pareto optimization may be useful in development to avoid it in production! After computing a set of Pareto-optimal answers, the user is left with the problem to draw conclusions from this set. Often, what users want is a single answer. This holds in particular when the “user” is a high-throughput pipeline. This calls for product operations such as ${*}_{\text {lex}}$ or ${*}_{\text {+}\lambda }$, as we used to provide in the past. But now, the designers of such a program can use Pareto optimization in the design stage to make a well-informed choice of the combination of objective functions eventually offered for production use.

Let us end this introduction with a word on Pareto optimization in higher dimensions than two. Pareto optimization can be defined over score vectors of any dimension. Here we deal only with two dimensions, providing the operator ${*}_{\text {Par}}$ that turns two scoring schemes *A* and *B* into their Pareto combination $\text {A} {*}\,\,_{\text {Par}}{}\,\, \text {B}$. This may suggest the idea that with $({A}\,\, {*}_{\text {Par}}{} \,\,{B})\,\, {*}_{\text {Par}}{}\,\, {C}$ we have Pareto optimization in three dimensions, and in four with $({A}\,\, {*}_{\text {Par}}{}\,\, {B}) {*}_{\text {Par}}{} ({C}\,\, {*}_{\text {Par}}{}\,\, {D})$. But No!, Pareto optimization is not modular in this sense. The prerequisite for ${*}_{\text {Par}}$ is that *A* and *B* optimize over a *total* order, while their Pareto combination optimizes over the partial order $(>_{A},>_{B})$. Hence, ${A}\,\, {*}_{\text {Par}}{} \,\,{B}$ is not admissible for further Pareto combinations. To arrive at higher dimensions of Pareto optimization, one must define a Pareto combination operator of flexible arity. Complexity of algorithms changes, and more sophisticated data structures, such as the quad-trees studied in [36] may come into focus. This is remains a challenge for future research.

## Endnotes

^a^For GAP-L experts: We supply the grammar with the algebra product $A*B$, where $\varphi _\text {A} = \varphi _\text {B} = id$, and add application of ${\mathbf{pf}} _{\varphi _\text {A},\varphi _\text {B}}$ via a semantic filter where appropriate.

^b^This is only a tendency—a final Pareto front of size *k* does not rule out intermediate results with Pareto fronts larger than *k*.

## Additional files

Additional file 1:
**Movie 1.** Transitions between the different structures in the Pareto front. Additional file 2:
**Movie 2.** Transitions between the different structures in the Pareto front. Additional file 3:
**Movie 3.** Transitions between the different structures in the Pareto front. Additional file 4:
**Movie 4.** Transitions between the different structures in the Pareto front. Additional file 5:
**Movie 5.** Transitions between the different structures in the Pareto front. Additional file 6:
**Movie 6.** Transitions between the different structures in the Pareto front.
