# Supplementary figures and images for: Pareto optimization in algebraic dynamic programming
Source: Algorithms Mol Biol. 2015 Jul 7;10:22. doi: 10.1186/s13015-015-0051-7 (PMC4491898; doi:10.1186/s13015-015-0051-7)

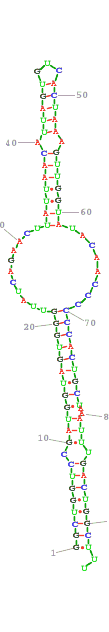

Supplement: Additional file 1: — Movie 1. Transitions between the different structures in the Pareto front. [file 13015_2015_51_MOESM1_ESM.gif]

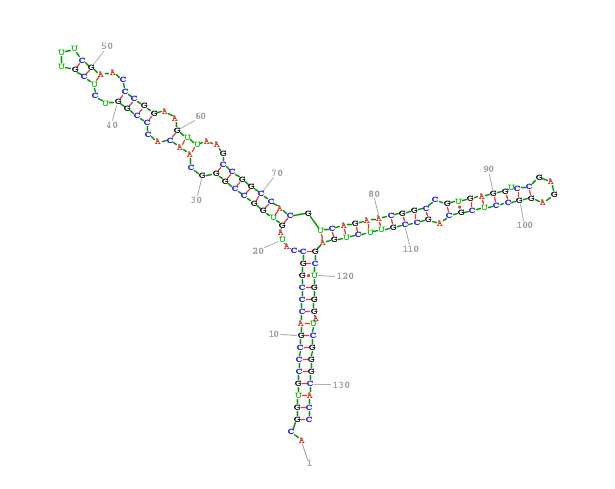

Supplement: Additional file 2: — Movie 2. Transitions between the different structures in the Pareto front. [file 13015_2015_51_MOESM2_ESM.gif]

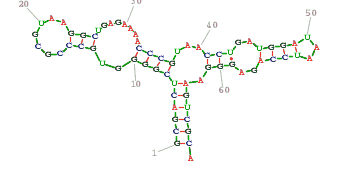

Supplement: Additional file 3: — Movie 3. Transitions between the different structures in the Pareto front. [file 13015_2015_51_MOESM3_ESM.gif]

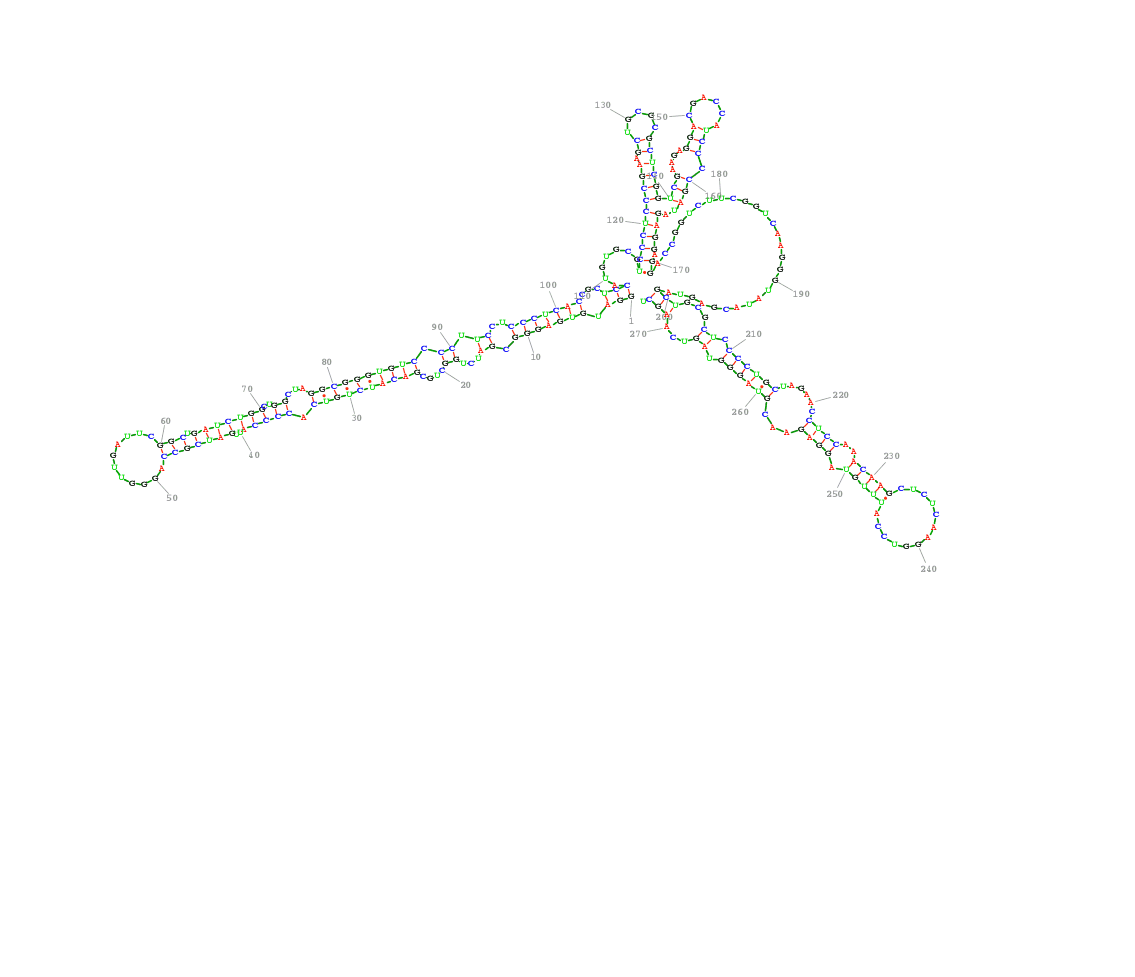

Supplement: Additional file 4: — Movie 4. Transitions between the different structures in the Pareto front. [file 13015_2015_51_MOESM4_ESM.gif]

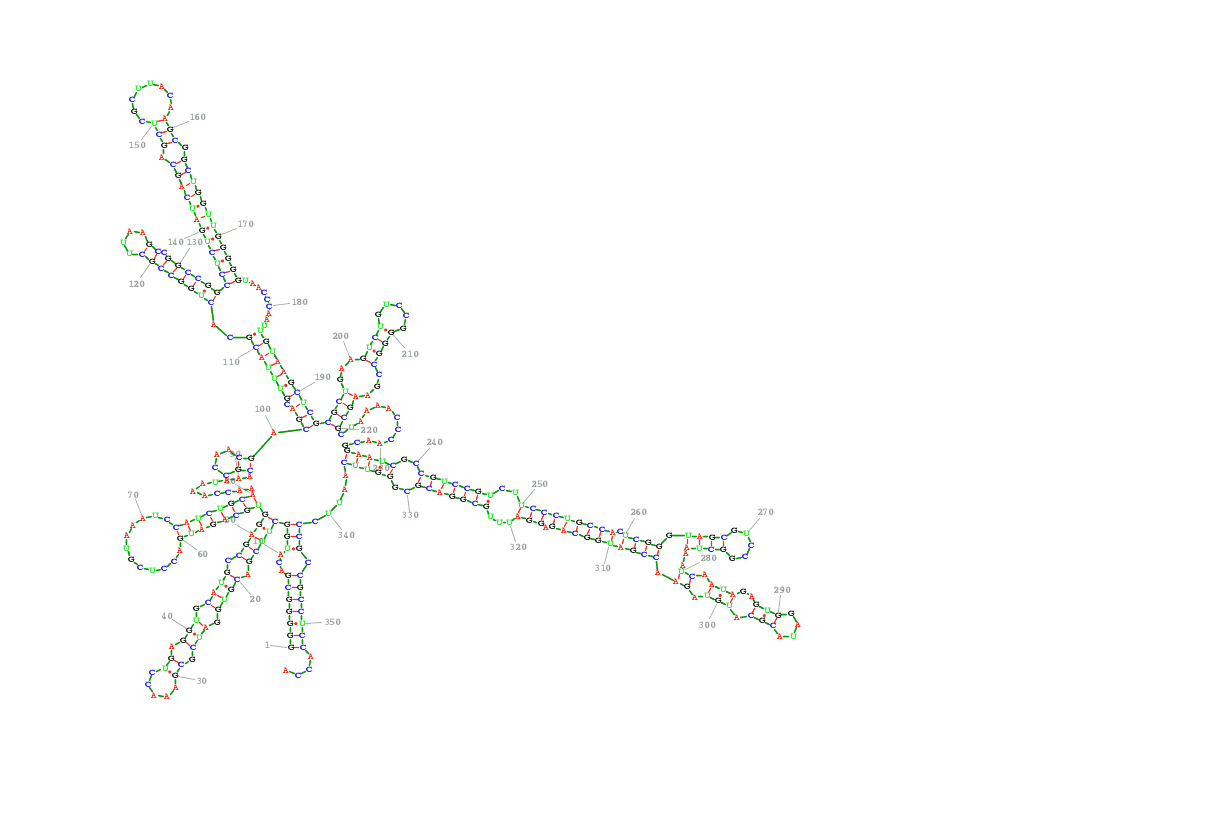

Supplement: Additional file 5: — Movie 5. Transitions between the different structures in the Pareto front. [file 13015_2015_51_MOESM5_ESM.gif]

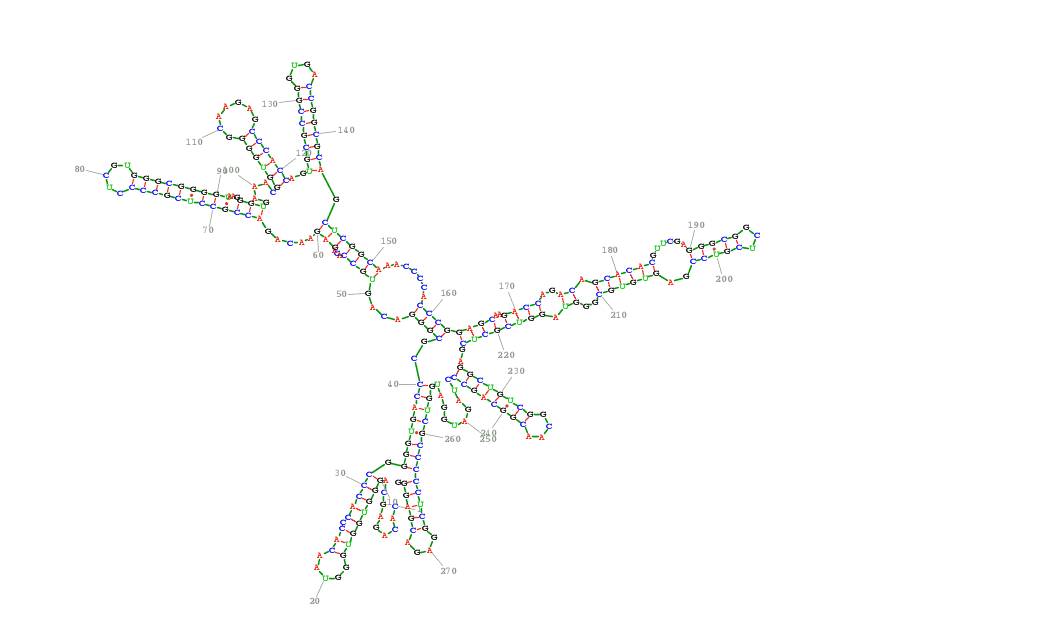

Supplement: Additional file 6: — Movie 6. Transitions between the different structures in the Pareto front. [file 13015_2015_51_MOESM6_ESM.gif]
